# Computational components of visual predictive coding circuitry

**DOI:** 10.3389/fncir.2023.1254009

**Published:** 2024-01-08

**Authors:** Stewart Shipp

**Affiliations:** Institute of Ophthalmology, University College London, London, United Kingdom

**Keywords:** prediction-error, predictive suppression, precision, visual hierarchy, gamma oscillations, cortical neurophysiology, laminar functionality

## Abstract

If a full visual percept can be said to be a ‘hypothesis’, so too can a neural ‘prediction’ – although the latter addresses one particular component of image content (such as 3-dimensional organisation, the interplay between lighting and surface colour, the future trajectory of moving objects, and so on). And, because processing is hierarchical, predictions generated at one level are conveyed in a backward direction to a lower level, seeking to predict, in fact, the neural activity at that prior stage of processing, and learning from errors signalled in the opposite direction. This is the essence of ‘predictive coding’, at once an algorithm for information processing and a theoretical basis for the nature of operations performed by the cerebral cortex. Neural models for the implementation of predictive coding invoke specific functional classes of neuron for generating, transmitting and receiving predictions, and for producing reciprocal error signals. Also a third general class, ‘precision’ neurons, tasked with regulating the magnitude of error signals contingent upon the confidence placed upon the prediction, i.e., the reliability and behavioural utility of the sensory data that it predicts. So, what is the ultimate source of a ‘prediction’? The answer is multifactorial: knowledge of the current environmental context and the immediate past, allied to memory and lifetime experience of the way of the world, doubtless fine-tuned by evolutionary history too. There are, in consequence, numerous potential avenues for experimenters seeking to manipulate subjects’ expectation, and examine the neural signals elicited by surprising, and less surprising visual stimuli. This review focuses upon the predictive physiology of mouse and monkey visual cortex, summarising and commenting on evidence to date, and placing it in the context of the broader field. It is concluded that predictive coding has a firm grounding in basic neuroscience and that, unsurprisingly, there remains much to learn.

## 1 Introduction

This review addresses functional diversity amongst pyramid neurons of the cerebral cortex – how far does current knowledge of anatomical and physiological characteristics permit their classification in terms of the computational roles envisaged by predictive coding theory? It extends a series examining the neural implementation of one theory in particular, the generalised predictive coding (gPC) scheme of Friston (2005) and Kanai et al. (2015): a generic comparison of the workings of visual and motor cortex (Shipp et al., 2013), closer scrutiny of the intrinsic circuitry of areas of visual cortex (Shipp, 2016) and an examination of the patterning of hierarchical, extrinsic connections between those areas (Shipp and Friston, 2023). Here, these structural principles of neural circuitry will serve as a framework in which to shift the focus to visual physiology. In the voluminous literature accumulated to date, how does the balance lie between confirmation or refutation of gPC – or toward blithe indifference?

There are, of course, several extant strains of hierarchical PC theories of cortical function, all tracing their ancestry from 19th century precepts that the brain should be capable of forming a predictive model of its environment, as opposed to simply registering passively the sensations it encounters (Helmholtz, 1860/1962). Some reiterate such heuristics (Keller and Mrsic-Flogel, 2018), whilst others have an explicit algorithmic basis, incorporating Bayesian statistics such that percepts are optimised by weighing momentary sensory evidence against prior knowledge and experience of the environment (Rao and Ballard, 1999; Friston and Kiebel, 2009; Spratling, 2016). Whatever their nature, all such theories posit two principal populations of computational units (i.e., neurons) reciprocally exchanging signals: so-called ‘prediction units’ and ‘error units’. Predictions flow down the hierarchy, their imperfections eliciting error signals in the reverse direction, acting to refine, or optimise predictions. Where this exchange is configured to traverse cortical areas at separate hierarchical levels, the assignment of computational roles to sub-populations of neurons is apodictic: backward projecting neurons must be prediction units, and forward projecting units must be error units (Mumford, 1992; Rao and Ballard, 1999; Friston, 2005; Bastos et al., 2012).

There are, however, significant numbers of neurons that lack such extrinsic projections: virtually all inhibitory interneurons, plus certain subclasses of pyramid neuron distributed unequally across cortical layers. The latter, ‘local’ pyramid neurons are rarely explicitly identified by anatomical study, as that requires reconstructing individual cell morphology (by intracellular dye injection or, classically, Golgi staining) to determine that no axonal process passes out of cortex into white matter. In primates, pyramid neurons in layer 2 and layer 4 (or layer 4C of primary visual cortex) are inferred to be local, on account of rarely, or never, being labelled by retrograde tracers injected into remote areas of cortex or any subcortical structure. The same does not apply to mice, where a continuous distribution of extrinsically projecting pyramid neurons occupies layers 2–6, precluding this indirect identification of local pyramids (Berezovskii et al., 2011). Analysis of pyramid neuron morphology (both extrinsic and local) reveals many subtypes of dendritic and axonal arborisation, varying in their relative profusion, laminar involvement and lateral spread; a rich tapestry, in other words, upon which to seek to map the computational architecture of gPC. A provisional scheme, or ‘template’, is outlined below. By way of introduction, gPC invokes six basic computational values. The exchange of prediction and prediction-error comes in two classes, relating to ‘*causes*’ (categorical variables such as colours, shapes, familiar items, gestures) and ‘*states*’ (dynamic temporal relationships between *causes*). Further, gPC models a probability distribution (or ‘expectation’) of both the mean and variance of these values, the latter being modelled by inverse variance, namely ‘precision’. As the estimate of precision is a second form of prediction, gPC specifies two distinct streams of backward messaging (Friston and Kiebel, 2009).

The provisional gPC ‘template’ circuitry offers some basic sanity checks with reference to known patterns of translaminar and lateral intrinsic connectivity, and links to what little is known of the generic physiology of particular layers, and of particular cell types. Building on these preliminaries, the focus of examination will then shift to certain select experimental paradigms cast in the mould of PC theories that aim to test or, at least, elucidate the mechanisms proposed for predictive processing.

## 2 gPC template construction

**Figure 1:**
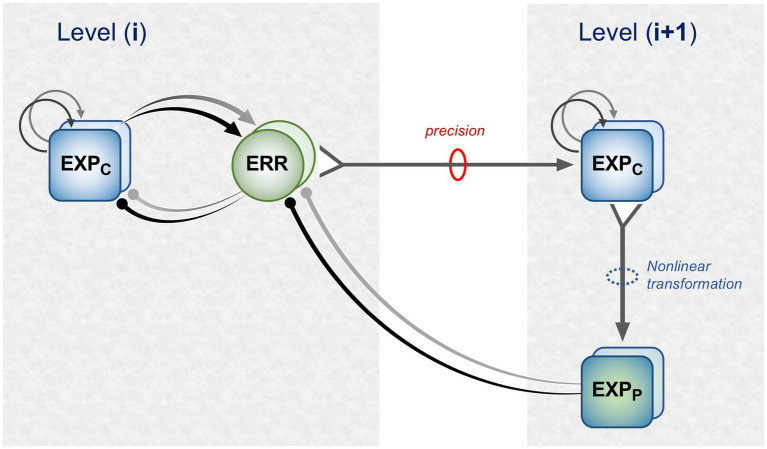
A simplified format for the computational architecture of the gPC algorithm. Dual expectation (EXP) and prediction error (ERR) units undertake parallel computations of *causes* and *states*; these are depicted as unit pairs in darker and lighter tones – but unspecified as to which is which, to reflect a commonality of computational strategy. The critical distinction, not rendered here, is that the exchange of messages between hierarchical stages, i.e., between levels (i) and (i + 1), applies only to the computation of *causes*; by contrast, the computation of *states* is discrete within each stage. Dual communications indicate transmission where *cause* and *state* values retain their separate identities; arrow terminals denote a positive effect, round terminals a negative effect. Single arrows (with forked tails) indicate transmission where both recipients in a pair of EXP units integrate both *cause* and *state* values (though independently and non-identically). Two types of EXP unit, ‘coding’ and ‘prediction’, are distinguished: the former (EXP_C_) encode causal features or feature-relationships at a certain level of abstraction, contingent on the hierarchical stage; the latter (EXP_P_) expresses a nonlinear transformation of the EXP_C_ value suitable for predicting a corresponding value of EXP_C_ expressed at the preceding stage (for *causes*), or the same stage (for *states*). Finally, forward (rightward) transmission from ERR to EXP units is regulated by precision, that controls the gain of ERR signals and hence their impact upon higher level processing. See Appendix A of Kanai et al. (2015) for the equations constituting this formulation of the gPC algorithm.

Figure 1 is a schematic, ‘neural’ rendition of the serial computational architecture of the gPC algorithm, configured from the perspective of error (ERR) units, with hierarchy ascending from left to right. Signals from ERR units, weighted by their precision, are directly processed by expectation units – specifically those labelled EXP_C_ – that represent specific features, or causes of sensory data (equivalent to what are termed ‘coding’, ‘value’ or ‘internal representation’ units in other accounts). The units labelled EXP_P_ express a nonlinear function of the EXP_C_ value, that serves as the prediction fed back to the ERR units, where it is compared with (i.e., subtracted from) the input ERR units receive from subordinate EXP_C_ units (shown at far left). All units are shown as pairs, corresponding to separate computation of values pertaining to *causes* and *states*; dual links show transmission retaining these separate identities, and single links where they are integrated. An important distinction, not rendered here, is that the computation of *states* is discrete, in that message passing is complete within a single stage of the computation (and recapitulated in subsequent stages); for *causes* this not so, as the unidirectional messaging from ERR to EXP_C_ and from EXP_P_ back to ERR takes place between serial stages of computation. See Figure 1 of Shipp (2016) for an alternative, fuller rendition of gPC architecture.

**Figure 2:**
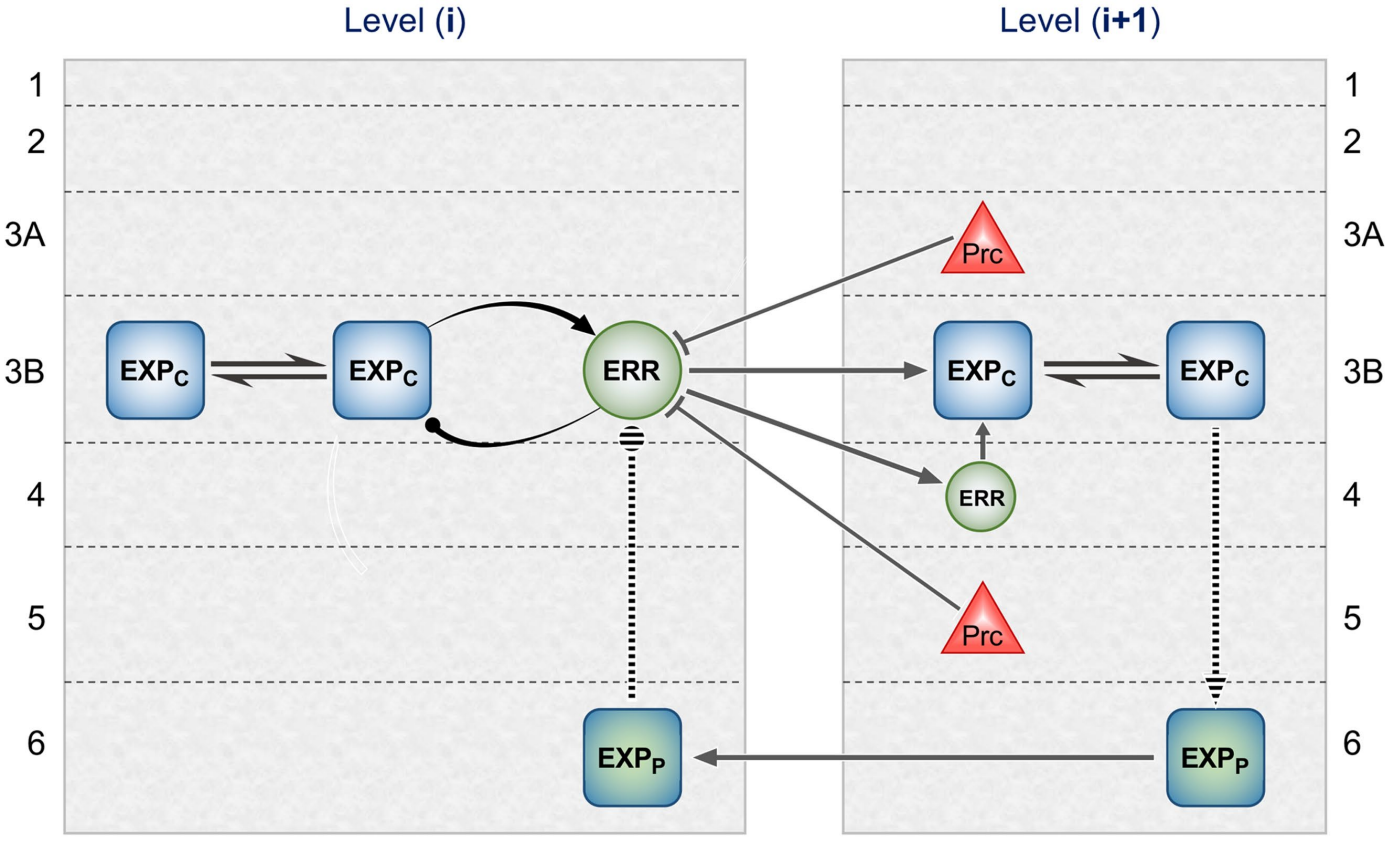
Model neural circuitry (or ‘template’) for gPC computational architecture. The template depicts elements of the intrinsic and extrinsic circuitry linking two areas at successive levels in mid-hierarchy (e.g., areas V2 and V4 of primate visual cortex). These are the minimum components (essentially, a ‘neural skeleton’) for implementing the gPC algorithm, further simplified by omitting the computational apparatus pertaining to *states*. The computational units comprise EXP_C_, EXP_P_ and ERR classes, as defined in Figure 1, plus ‘Prc’ units, encoding values of precision. EXPc units in layer 3B at both levels can be interpreted as intrinsic pyramids; for diagrammatic reasons each shows just a subset of a common connectome. The EXP_P_ unit in layer 6 at the higher level is a FB projection neuron; the EXP_P_ unit in layer 6 at the lower level is an intrinsic pyramid, receiving FB input. The extrinsic ERR unit, shown in level (i), is an FF projection neuron; the ERR unit in layer 4 of level (i + 1) is an intrinsic, small pyramid (or ‘granule’ cell), characteristic of this layer. Prc units in layers 3A and 5 are also FB projection neurons. Solid arrowed lines indicate direct monosynaptic excitatory connections; Prc unit outputs with arc endings are modulatory (N.B. contacting apical dendrites in superficial layers, not the cell body in layer 3B). Ball endings indicate inhibitory transmission through an intervening interneuron. Dashed connectors leading to, or from EXP_P_ units in layer 6 denote translaminar transmission of unspecified nature (see text for further details).

Figure 2 maps this scheme upon the basic elements of intrinsic and extrinsic neural circuitry linking two cortical areas, though simplified by omitting the computational units relating to *states.* It is referred to as a ‘template’ to indicate its provisional and rather elementary nature in comparison to the known complexity of cortical wiring. The format is slightly more abstract than previous renditions of the gPC template, but relies upon the same analysis of circuit details, largely drawn from primate visual cortex (e.g., the relationship between areas V2 and V4). Note that V1 is less suitable for a generic model, on account of its unique laminar structure and the fact that, being at the base of the hierarchy, it does not issue back connections to a subordinate cortical area.

### 2.1 Forward pathway via layers 4 and 3B

ERR output units correspond to feedforward (FF) pyramid neurons, that are concentrated in layer 3B (FF neurons also occur in layers 5 and 6, and some are scattered above layer 3B); they project principally upon layer 4 but also, less densely, above layer 4 (Rockland and Pandya, 1979; Lund et al., 1981; Zeki and Shipp, 1989; Rockland, 1992a). Thus synaptic contact with pyramid neurons of layer 3B seems likely, either upon a cell body or upon its basal dendrites ramifying within layer 4. These neurons would be interpreted as intrinsic EXP_C_ units, subserving the minimal, disynaptic chain through an area specified by gPC; the second synapse is upon an FF ERR unit, again in layer 3B. Following Bastos et al. (2012), the smaller local pyramid neurons of layer 4 are interpreted as intrinsic ERR units, the first stage of a putative tri-synaptic chain through the area. Existing theories of the function of the granular layer 4 of cortex – amplification, and/or interpolation of afferent signals – are not incompatible with the gPC model. Empirical verification of the minimal number of links in an FF chain through an area is vanishingly scarce (across systems and species): but, in primates, the pathways from magno- and parvo-cellular LGN through V1 to V5 and V4 have, respectively, been demonstrated to be disynaptic and trisynaptic (Ninomiya et al., 2011); the latter is confirmed by cell-morphological analysis of intrinsic circuitry, in that only local, but not FF pyramid neurons of the superficial layers of V1 were found to receive direct contacts from parvocellular relay neurons of layer 4Cb (Sawatari and Callaway, 2000). Hence these two pathways through V1 serve as analogues for the di-and trisynaptic chains devised by the gPC template for traversing areas of extrastriate cortex.

Each iteration of the computation of expectation combines the current expectation with prediction error arising from the previous iteration. Thus reciprocal connections between EXP_C_ units in layer 3B stand in for the recurrent inputs shown in Figure 1. This is supported by evidence of preferential connectivity amongst superficial pyramid neurons of similar feature selectivity (Livingstone and Hubel, 1984b; Yoshioka et al., 1996; Hu and Roe, 2022), particularly co-axially aligned orientation tuning (Bosking et al., 1997; Sincich and Blasdel, 2001; Iacaruso et al., 2017) and the fact that layer 3 is found to demonstrate a high density lattice of extended lateral connections in most (primate) areas, without known exceptions (Lund et al., 1993). By contrast, the negative feedback loop between EXP_C_ and ERR units should be formed by more localised connections. As previously discussed (Shipp, 2016), somatostatin expressing interneurons have the appropriate characteristics for the feature selective inhibition of EXP_C_ units by ERR units; PV expressing interneurons (‘basket cells’, specifically) have longer range axonal arbours and are better suited for the role of gain control within the extended EXP_C_ network. Evidence for the exclusion of FF ERR units from a large-scale lateral network is provided by the study of Ichinohe et al. (2012) who performed a two-stage procedure, using three distinct retrograde tracers, to examine connections between areas TEO and TE in monkeys. Stage 1 visualised, *in vivo*, several separate patches of FF neurons in area TEO projecting to the site of a single injection of (red) tracer in area TE. In stage 2, two other tracers were injected in TEO, one (green) directly into a patch of red labelled cells (i.e., amidst identified TE-efferent cells), the other (gold) at a site external to those patches – giving rise to two further patchy systems of labelled cells, this time revealing networks of intrinsic connections within TEO. These three sets of labelled cells formed just two distinct networks, one formed by heavily overlapping patches of red and green labelled cells, the other by separate gold-labelled patches interdigitated between them (− presumptively, the red/green patches corresponding to a set of columns with featural selectivity congruent with that of the injection site in area TE, and the gold patches representing an incongruent feature). Crucially, despite the co-distribution of the ‘red’ and ‘green’ networks, double-labelled (green/red) cells were extremely scarce, apart from the immediate vicinity of the green tracer injection, i.e., within the target patch of red-labelled TE efferent cells, where they were common. The outcome was thus entirely consistent with the gPC template of Figure 2 in showing that, as the authors themselves concluded, FF neurons’ axon collaterals make only short-range intrinsic connections and that longer range intrinsic connections are only formed by neurons lacking a FF projection (Ichinohe et al., 2012).

Arguably, of course, that study does not conclusively demonstrate that the larger scale intrinsic network of putative EXP_C_ units in area TEO purely comprised local pyramids, as the neurons concerned might have formed extrinsic connections to sites anywhere in the brain outside area TE. As noted above, local pyramids are rarely explicitly identified – although one prominent type has been documented in layer 3B of area V2 (Lund et al., 1981). However, to dwell on this point, it is worth noting that the template only precludes EXPc units from contributing to the subsequent stage of gPC computation (i.e., to a higher, more abstract level of featural processing). It does not outlaw outputs to external systems utilising visual information at a certain level of abstraction, e.g., to medial temporal lobe/hippocampus for the purposes of associative memory encoding; or, to the caudate/putamen input stage of the subcortical basal ganglia loop, to learn the sensory context of rewarded actions. Indeed, EXP rather than ERR units are the natural candidates for such an ancillary role.

### 2.2 Backward pathways from superficial and deep layers

Anatomically, feed-backward (FB) pathways have bipolar origins and bipolar terminations (Markov et al., 2014; Shipp, 2016). The superficial stream, projecting mainly from layer 3A, focuses upon layer 1. The deep stream, originating in layers 5 and 6, terminates more equally between the superficial and deep layers; it also has greater range, in that it may span several levels of hierarchy. As noted above, the gPC algorithm provides for two forms of backward messaging, prediction and precision. The gPC template of Figure 2 provisionally allocates precision FB units to layers 3A and 5, and prediction FB units to layer 6. Precision FB units should be capable of regulating the gain of FF ERR units by virtue of contacting their apical dendrites arborising in layer 1 – a process known as ‘apical amplification’ (Phillips, 2017). By contrast, prediction (EXP_P_) units should be capable of a more driving influence upon their targets – also nominated as EXP_P_ units – in order to govern the information content of their signalling; this would be mediated by terminating upon perisomatic dendrites, as better achieved by the deep component of the backward projection to layers 5 and 6. Evidence consistent with these assignments has recently been obtained for looped connections between V2 and V1 in primates: specifically, the laminar distribution in V2 of FB neurons determined to make direct synaptic contact upon the FF neurons of V1 that project to V2. This subpopulation of FB neurons was prominent in layers 3A and 5 of V2, but largely absent from layer 6 (Siu et al., 2021). By contrast blanket retrograde tracing, that detects sources of FB input to *all* cells in V1, shows a higher frequency of FB neurons in layer 6 than layer 5 of V2 (Markov et al., 2014). If these layer 6 FB neurons of V2 do not directly contact superficial FF cells, they must be inferred to focus their backward projection upon the deep layers of V1. The gPC template takes this anatomical pattern to be conserved between higher extrastriate areas.

The nature of the translaminar circuitry connecting the EXP_P_ units in layer 6 to layer 3B is less well specified, with several possibilities at both the higher level area (from layer 3 to 6) and the lower level area (from layer 6 to 3). In the higher level area, the pathway leading to FB EXP_P_ units must compute a nonlinear transform of combined output from both *cause* and *state* EXP_C_ units (as indicated in Figure 1). Studies of translaminar connectivity in extrastriate areas V2, V4 and TE concur that a direct output from layer 3 to layer 6 is present, but lighter than that to layer 5, which itself has output to layer 6 (Yoshioka et al., 1992; Levitt et al., 1994; Fujita and Fujita, 1996). Thus there can be both direct and indirect (mono- and di-synaptic) routes to contact peri-somatic dendrites of layer 6 pyramids. A third route is direct transmission to rising dendritic arborisations of layer 6 pyramids within layer 5, or within layer 3B itself (Lund et al., 1981; Yoshioka et al., 1992). These possibilities might be viewed as complementary, rather than mutually exclusive. Turning to the lower level area, recipient EXP_P_ units here are inferred to be local pyramids with superficial axon arborisations reaching to layer 3. Several types of pyramid neuron matching this morphological description have been described in layers 5 and 6 of V1 (Callaway and Wiser, 1996; Wiser and Callaway, 1996, 1997; Briggs and Callaway, 2001). They would then transmit to FF ERR_C_ units via a local interneuron [and, again in V1, several morphologically distinct candidates are available (Lund and Wu, 1997)]. Direct inhibitory transmission is also possible – specifically from layer 5 interneurons of V1 (Lund et al., 1988) – though less favoured, in view of the fact that interneurons are less numerous than pyramid neurons. The known anatomical picture for extrastriate cortex is far less detailed; columnar transmission from layer 6 to layer 3 is reported, though the specific cells of origin were not determined (Yoshioka et al., 1992; Levitt et al., 1994; Fujita and Fujita, 1996).

Taking an overview, it is remarkable – and encouraging – that an information processing theory with non-biological origins (Bayesian statistics and machine learning) should map quite so adroitly upon cortical circuitry: that FF and FB projection neurons do form separate populations, with scant traces of hybridisation (Vezoli et al., 2021; Shipp and Friston, 2023); that the duality of backward messaging can be accommodated by the bipolar organisation of FB pathways; and that intrinsic neurons exist with appropriate lateral and translaminar connections to fulfil the requisite links between FF and FB systems. It ratifies much of the basic framework, if little of the baroque ornamentation of cortical architecture. But that is foundation enough to begin to consider how these ideas translate into characteristics of physiological function.

## 3 Generic physiology

At first sight, it can seem bizarre that half a century of cortical neurophysiology fails to arbitrate the pivotal question – is gPC a viable theory of cortical function? But the grounds for that inadequacy are not so cryptic: reportable ‘findings’ typically characterise select populations of neurons, and common criteria for defining sub-populations of neurons effectively amalgamate the notional gPC functional classes. Columnar sets of EXP, ERR and Prc units likely share similar featural selectivity (e.g., contour orientation, border ownership, motion direction, retinal disparity, higher forms of shape or facial configuration, etc). They might be distinguished by rather more subtle attributes of spatiotemporal context sensitivity or spiking dynamics that do not immediately suggest themselves to investigators seeking objective criteria for parcellation of collective data. Anatomical data-partitions by area or by, say, cytochrome-oxidase module, miss the boat for similar reasons. Partition by cortical layer, as we shall see, offers better prospects: but then, studies undertaking this exercise with the necessary rigour are few and far between. For our purposes, physiological characterisation ideally pertains to a neural sub-population determined by cell morphology, or extrinsic projection type, or genetic phenotype; these are presently facilitated by genetic manipulation of mouse lines, but not yet primate.

This is not to dismiss the ‘generic’ neurophysiological literature (i.e., for present purposes, that which does not explicitly address predictive processing). There is much that is insightful: findings that are consonant with gPC theory, or that expand concepts of how the cortex might implement the gPC algorithm. Some have been reviewed previously (cortico-geniculate transmission, apical dendritic function, lateral connectivity amongst neurons with common feature selectivity) and will not be recapitulated here (Shipp, 2016). The following examples augment this (far-from-encyclopaedic) synopsis.

### 3.1 Simple and complex cells

Following their discovery in V1 of cats and monkeys, Hubel and Wiesel’s satisfying intuition that ‘complex’ receptive fields are formed by converging inputs from cells with ‘simple’ receptive fields has stood the test of time. Paired recordings from the two cell types in cat V1 concur with this hierarchical theory, showing that complex cells are driven by monosynaptic inputs from pools of synchronously spiking simple cells, with no evidence for monosynaptic excitation in the reverse direction (Alonso and Martinez, 1998; Yu and Ferster, 2013). Translating this generic finding to the monkey, the point of note here is that single cells of layer 4B of V1 identified by antidromic activation to project to area V5 were found to be highly direction selective with complex receptive fields – all six of them (plus six in layer 6), a rare example of physiological characterisation of a certified projection type (Movshon and Newsome, 1996). These complex cells, anatomically identified as large stellate cells (Shipp and Zeki, 1989; Nassi and Callaway, 2007), must be fed by simple cells, some of them in layer 4Ca (Blasdel and Fitzpatrick, 1984; Livingstone and Hubel, 1984a; Hawken et al., 1988; Gur et al., 2005). The latter are intrinsic, small spiny stellate cells, receiving magnocellular geniculate input and projecting upon layer 4B (Yabuta and Callaway, 1998). As noted above, the implied minimal disynaptic route from LGN to V5 via layers 4Ca and 4B has been empirically confirmed by trans-synaptic retrograde tracing with rabies virus (Ninomiya et al., 2011). Detailed statistical modelling of data recorded from this system again weighs in favour of a purely excitatory feedforward model (Lochmann et al., 2013), whereby direction-selective complex cells are fed by pools of similarly direction-tuned simple cells, inheriting their directional properties but creating invariance for spatial phase (i.e., the typical ‘complex’ property of uniform sensitivity to both light and dark contrast across the entire receptive field).

From the perspective of gPC, the circuitry dictates that the simple cells of 4Ca must be EXP units, and the output complex cells of layer 4B must be ERR units. Nothing in the above analysis validates the implied ‘expectation’ or ‘error’ functionality, but two conclusions (or considerations) do present themselves. The first, quite simply, is that all this evidence concurs with the unidirectional excitatory contact from EXP units to ERR units shown by the gPC template (Figure 2). The second informs the nature of predictive processing. The prediction fed back from V5 to the prospective ERR unit in layer 4B (that might arrive via layer 6 of V1 or, in this unique system, terminate directly within layer 4B itself) should match what that unit is capable of signalling, namely the direction of motion of an object or texture irrespective of its spatial phase or contrast. Hence, it does not precisely match the nature of any individual EXP signal forwarded by a simple cell. Whilst those forward EXP signals to the ERR unit are spatial phase or contrast sensitive (Lochmann et al., 2013), the backward predictive signal is not; the latter predicts a population property of the of EXP units, that the ERR unit abstracts from its inputs. There is little reason to suppose that such a pooling process – a computational step operating in the forward pathway from EXP to ERR units – is unique to this early level of the motion pathway. The latter is merely the sole system where the requisite anatomical and physiological findings coalesce sufficiently to discern it.

### 3.2 Precision

Precision, by regulating the impact of ascending error signals upon higher level representations, operates the Bayesian balance between incoming sensory data and prior expectation in determining what we actually perceive, or do, depending on the brain system in question (Adams et al., 2013). Prior expectation, of course, is multifactorial, drawing upon lifetime experience allied to knowledge of the current environmental and behavioural context. And, just as representation, and expectation, grow more abstract across the cortical hierarchy, so too does the nature of precision. At the lowest level precision predicts the reliability of sensory data, and can be estimated from the magnitude of the accompanying prediction errors. This optimises the veracity of perception. Higher levels progressively incorporate behavioural context, or in other words the expected relative utility of various forms of sensory data. Thus precision can be fairly equated with attentional mechanisms in modulating brain activity. In the visual system attentional effects permeate just about every structure outside the retina. So too, precision might operate through multiple neural mechanisms: proposed candidates include the various neuromodulatory systems, corticocortical feedback, and subcortical loops. For example, see Shipp and Friston (2023) for a first account of how prediction error pooled across feature modality and circulated through the superior colliculus and pulvinar might generate precision relating to spatial attention. Here, an inferred precision network in the superficial layers of area V2 is examined in relation to a potential role in feature attention.

This proposal, relating to feature binding and attention, derives from the finding that neurons with bimodal tuning – dual selectivity for both chromatic and spatiotemporal features (orientation and/or direction selectivity) – are significantly more frequent amongst the superficial and deep layers of V2 that receive backward projections (Shipp et al., 2009). With respect to the current layer terminology, bimodality is a conspicuous feature of layers 2, 3A, 5 and 6 of V2, but not of the forward pathway through layers 4 and 3B, where unimodal neurons are predominant. It was recognised that, being so positioned, bimodal neurons would be capable of mediating feature attention effects conveyed via backward pathways (Maunsell and Treue, 2006; Liu, 2019) – although, being obtained under the feedback-attenuating influence of anaesthesia, the bimodal property itself was likely attributable to convergent processing of unimodal forward input from V1. For instance there are certain cross-modal psychophysical effects demonstrable with bichromatic dot displays, whereby selective attention to one component colour determines the direction of a motion aftereffect (Sohn et al., 2004), or enhances sensitivity for detection of coherent motion (Croner and Albright, 1997). These are challenging to explain when area V5 (Gegenfurtner et al., 1994; Thiele et al., 1999; Barberini et al., 2005), and its directionally selective sources in V1 and V2 (Movshon and Newsome, 1996; Shipp and Zeki, 2002; Horwitz and Albright, 2005), are all well characterised to lack chromatic selectivity.

**Figure 3:**
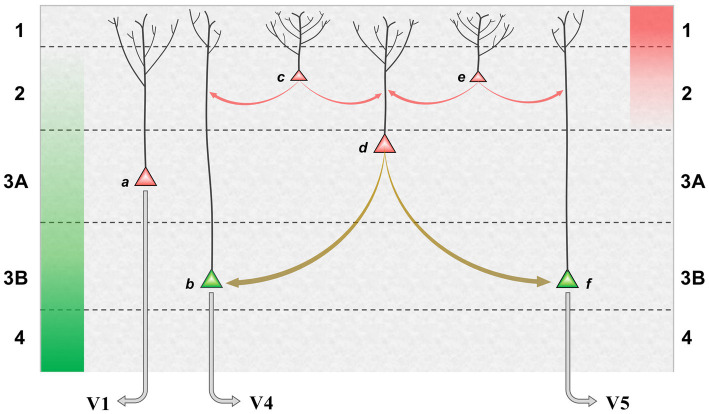
Model of a precision network in superficial layers of area V2. Green and red columns, at far left and right, indicate laminar range and intensity of FF and FB afferent systems: green, superficial axon terminations of layer 4 granule cells receiving input from V1; red, axon terminations of backward projections from areas such as V4 and TEO. Pyramid neurons credited as precision units (red) or ERR units (green) are shown at various depths, those lying more superficially bearing more profuse apical dendritic arborisation within layer 1 (basal dendrites not shown); EXP units are omitted. (a) FB pyramid in layer 3A identified to project to V1. (b,F) FF pyramids in layer 3B identified to project to either area V4, or V5 (these cells are known to occur in segregated clusters). (c,e) Local pyramids in layer 2, inferred to make lateral contacts upon neurons throughout layers 2 and 3. (d) A ‘bridge neuron’ – with indeterminate status as local or FB pyramid, but classified physiologically by bimodal feature tuning and conjectured to contact FF pyramids in layer 3B. Red arrows indicate lateral communication with predominant origin in backward (precision) processing; olive arrows indicate lateral communication originating from integration of forward and backward processing. It is not known precisely how lateral connections distribute across peri-somatic, basal or apical dendritic cell compartments; the arrows indicate the target neuron, not the point of contact.

The proposed mechanism, in which bimodal neurons act as ‘bridge neurons’ between unimodal outputs, can now be set in the broader context of a precision network in V2. Figure 3 shows a model of the superficial layers combining pyramid morphology obtained by Golgi staining (Lund et al., 1981) with knowledge of connectivity. FF signals, relayed from layer 4, are maximal in layer 3B and peter out toward layer 2; FB signals are focussed upon layer 1, diminishing in intensity through layer 2. Inhabiting this counter-stream architecture are pyramid neurons whose dendritic arbors enable differential sampling of FF and FB signals. The basal dendrites of deeper pyramids, in layer 3, receive FF signals whilst their apical dendrites can sample FB signals. By comparison, pyramid neurons lying more superficially are observed to have more profuse apical arborisations in layers 1 and 2. Of note, a corresponding continuum has been quantitatively documented in layer 2/3 of mouse V1, where pyramid neurons lying more superficially receive progressively less input from layer 4, and have progressively broader apical dendritic trees (Weiler et al., 2023). In macaque V2, layer 3B houses the forward pathway, posited to comprise EXP and ERR pyramid neurons. Layer 3A, by contrast, is the primary source of the superficial backward pathway to V1, still well within the laminar zone of afferents from layer 4. The pyramid neurons here are depicted as precision units, estimating sensory data reliability from ascending error signals, but likely also collecting modulating influences from layer 1. More superficially, there are increasing numbers of neurons inferred to lack extrinsic output (confirmed, at least in principle, by a solitary Golgi reconstruction of a V2, layer 2 pyramid with axonal arborisation confined to this layer (Valverde, 1978)). These are increasingly dominated by intralaminar and FB inputs – perhaps exclusively so if the entire dendritic arborisation, not merely its apical component, accesses FB drive. All of these neurons contribute extensive axon collaterals to a horizontal network, noted to be densest in layer 3B. Thus intrinsic superficial pyramid neurons may also be interpreted as precision units, tasked with local processing of FB signals prior to exerting a modulating influence upon elements of the forward pathway.

Our so-called bridge neurons were found throughout layers 2 and 3A of V2. Being defined by the physiological property of dual-tuning, their identity, connection-wise, as extrinsic or local pyramids is unknown. However, they are proposed to make intrinsic connections with separate clusters of FF neurons (ergo ERR units) in layer 3B transmitting signals relating to form/colour, or motion, respectively, to areas V4 and V5. The functional logic of the proposed bridging mechanism can be illustrated with respect to the enhancement of sensitivity to coherent dot motion – observed both psychometrically (Croner and Albright, 1997) and neurometrically in area V5 of macaque monkeys (Croner and Albright, 1999) – when the subset of coherent dots, i.e., those moving in a uniform direction, is made salient in colour with respect to the co-extensive cloud of dots moving incoherently. Suppose the coherent dots are red and move upward. The display elicits feature attention to red that, in physiological terms, modulates the responsiveness of all red-sensitive visual neurons. Amongst bridge neurons tuned to various combinations of colour and direction of motion, those selective for ‘red-up’ will experience optimal forward drive and backward apical amplification. This influence can, in turn, be communicated to FF output neurons with matching, albeit unimodal tuning. In this example, the amplification of ‘up’ direction-selective neurons efferent to area V5 is the crucial outcome, effectively raising sensitivity for coherent motion detection in comparison to a monochromatic display. Corroboratory findings from human fMRI indicate that V2 plays a pivotal role in mediating a colour-motion mis-binding illusion (Zhang X. L. et al., 2014).

Are there further characteristics of layer 2 neurons, interpretable as adaptations toward processing of backward input? Unfortunately, suitable studies of superficial layer physiology are few and far between, certainly in the primate literature. In V1, the receptive fields of layer 2 neurons were characterised as larger than those of layer 3 but less precisely tuned for spatiotemporal features; chromatic properties were not assessed (Gur and Snodderly, 2008). The recordings were made in alert but passively behaving subjects, and could yet be consistent with an intrinsic modulatory role, in contrast to FF neurons in layer 3 with better capability to transmit specific, focal image features. Layer 2 also showed significantly higher spontaneous activity, perhaps consonant with a couple of electrical characteristics of neurons in layer 2 of mouse V1 – higher membrane input resistance and longer time constants – as noted by Weiler et al. (2023). Whilst, therefore, FB precision signalling finds an initial, if tenuous foothold in generic physiology, the nature of FF error signalling is more securely rooted in well-documented aspects of cellular electrophysiology and neural dynamics (Section 3.3).

### 3.3 Gamma oscillations

Neural oscillations of varied frequency are attributed multiple roles in perception and cognition, facilitating signal processing and transmission. Gamma rhythms (30-40 Hz and above) are credited with regulating effective connectivity or, in other words, mediating selective communication within a pluripotent anatomical network (Fries, 2005; Akam and Kullmann, 2014; Buzsaki and Schomburg, 2015; Fries, 2015). Gamma is also characteristic of FF transmission, by virtue of asymmetric causality in hierarchical systems (van Kerkoerle et al., 2014; Bastos et al., 2015b; Michalareas et al., 2016; Ferro et al., 2021), and being more strongly expressed in superficial cortex where FF neuron density is maximal (Buffalo et al., 2011). Since, in neural models of gPC, FF connections are associated with error signalling, so too must gamma oscillations. This is ratified empirically by behavioural paradigms designed to evoke a prediction error when conditioned expectation is violated by a ‘surprising’ stimulus: transient gamma enhancement is observed, both in EEG/MEG study of human subjects (Bauer et al., 2014; Brodski et al., 2015; van Pelt et al., 2016), and invasive recordings from monkeys (Bastos et al., 2020; Esmailpour et al., 2022) as reviewed below (Section 4).

Are gamma oscillations specific to ERR unit activity, or common to all superficial layer neurons? Clarification of this question, at least in respect of V1, is afforded by the identification of a sub-class of excitatory cell characterised by a narrow spike waveform and high propensity for burst-firing (Onorato et al., 2020). These ‘bNW’ (bursting, narrow-waveform) neurons comprised 30% of neurons recorded in the superficial layers of macaque V1, as distinct from two other classes, broad-spiking excitatory neurons and non-bursting narrow spiking neurons (inferred interneurons). The crucial observation is that, of these three classes, bNW neurons showed the tightest phase-locking to the local gamma cycle (determined from concurrent recording of the local field potential), and their spikes occurred fractionally earlier than the spikes of interneurons, consistent with computational modelling of gamma genesis through reciprocal E-I interactions (Buzsaki and Wang, 2012). The population of bNW neurons thus has the numerical frequency and spiking characteristics to serve as ERR units, and anatomically should correspond to the superficial FF cells that are densest in layer 3A of V1 (Rockland, 1992b). Macaque and cat V1 are similar to each other (and dissimilar to mouse V1) in generating uniquely strong gamma rhythms upon visual stimulation, in comparison to other visual areas. Importantly therefore, cat V1 is known to have an equivalent burst-spiking class of pyramid neuron – ‘chattering cells’ – further characterised by prominent gamma oscillations of membrane potential upon visual stimulation, as recorded intracellularly (Gray and McCormick, 1996). Notably, cells of this type were restricted to layer 2/3 of cat V1 where the great majority of FF neurons are located (Ferrer et al., 1988; Shipp and Grant, 1991), and subsequent morphological analysis by cell dye-injection indeed revealed axons passing into white matter.

Gamma oscillations can additionally be seen as a mechanism subserving precision, in that they are instrumental in governing effective connectivity between cortical areas – according to a theoretical construct known variously as ‘communication through coherence’ or ‘routing by synchrony’ (Fries, 2005; Kreiter, 2006). This is evidenced by simultaneous dual recordings from areas V1 and V4. Two studies followed a similar behavioural strategy of cueing attention to one or other of a pair of stimuli that were far enough apart to activate separate groups of neurons in V1, but close enough to fall together within the larger receptive fields of V4 neurons (Bosman et al., 2012; Grothe et al., 2012). A well-replicated finding in this scenario is the ‘shrinking receptive field phenomenon’: faced with such dual stimulation, V4 neurons respond selectively to whichever is attended, such that a stimulus whose colour or form normally elicits a strong response will fail to do so when a less preferred stimulus is attended (Moran and Desimone, 1985; Luck et al., 1997; Reynolds et al., 1999). In neural terms, attention appears to gate which source of afferent input from V1 is able to communicate with V4. The studies in question measured gamma coherence (i.e., a consistent phase relationship over time between gamma cycles operating in V1 and V4) and found that it was substantial with respect to the locus of the attended stimulus in V1, and negligible with respect to the other (Bosman et al., 2012; Grothe et al., 2012). Given such sustained coherence, spikes arising during the excitatory phase of one cycle might be timed to arrive at the period of maximal excitability of the other. The conventional metric for effective connectivity in either direction is granger causality (GC; Friston et al., 2014), obtainable as a spectrum of transmission magnitude against oscillation frequency. And, applying this metric to bidirectional transmission between V1 and V4 across trials with attention alternating between the two sites in V1, the most notable observation is a peak in GC for gamma frequencies (60–80 Hz) in the attended condition, for FF transmission from V1 to V4 (Bosman et al., 2012).

The neural mechanisms underpinning transareal coherence remain poorly understood. Of note, the recordings were local field potentials, obtained by intracortical electrodes in one case (Grothe et al., 2012) and by sub-pial contacts in the other (Bosman et al., 2012) – both susceptible to activity from a broader population of neurons than those directly emitting or receiving transcortical signals. Furthermore, direct connections between areas V1 and V4 are relatively sparse (Yukie and Iwai, 1985; Steele et al., 1991; Nakamura et al., 1993; Ungerleider et al., 2008), and the bulk of signal transmission is likely to relay via area V2, itself known to engage in gamma coherence with V1 (Roberts et al., 2013). Conceivably, transareal coherence at gamma frequencies depends upon a gamut of precision mechanisms. Within V1, separate stimulation sites should desynchronise in order to allow selective coherence with one or the other; cholinergic modulation, known to facilitate visual attention, has been implicated in mediating such local desynchronisation in occipital cortex and area V1 in particular (Pinto et al., 2013; Chen et al., 2015; Sajedin et al., 2019). This might also instantiate laminar-specific modulation of inhibitory mechanisms (Katsanevaki et al., 2023). Acting more broadly, subcortical re-entrant loops, operating from fronto-parietal control centres via colliculus and pulvinar have long been proposed to regulate and coordinate the transareal propagation of oscillations (Lopes da Silva et al., 1980; Shipp, 2003, 2004; Bourgeois et al., 2020) and evidence to this effect is beginning to accumulate (Saalmann et al., 2012; Fitzgerald et al., 2013; Zhou et al., 2016; Bonnefond et al., 2017; Quax et al., 2017; Eradath et al., 2021). A significant experimental obstacle is the requirement for simultaneous recording from multiple, precisely targeted cortical and subcortical sites. This Section concludes with a final study – ‘Top-down Beta Enhances Bottom-Up Gamma’ (Richter et al., 2017) – that is particularly notable for examining coordinated activity from three separate cortical sites and tying together several of the precepts of top-down precision regulation as advanced above.

The background to this work is the original proposition that top-down signals (in a cognitive sense) exploit coherence in the beta range of frequencies (13–30 Hz) and might act to modulate bottom-up gamma signals (Wang, 2010). Subsequent systematic evaluation of the anatomically validated visual hierarchy in macaques (Bastos et al., 2015b), and its presumed human equivalent (Michalareas et al., 2016) established causal asymmetry in oscillatory transmission: gamma GC is prevalent in the forward direction and beta (or alpha/beta) GC in the backward direction. Notably, such a decrement in frequency from forward to backward transmission is inherent in the gPC algorithm, which dictates that expectation/prediction units must show low-pass dynamics as a consequence of frequency attenuation in assimilating cumulative prediction error (Friston, 2008; Bastos et al., 2015a). The study of Richter et al. (2017) utilises the same electrocorticographic data mentioned above (Bosman et al., 2012; Bastos et al., 2015b) and focuses upon area 7A in addition to V1 and V4 – area 7A being a parietal area implicated in shifting spatial attention (Robinson et al., 1995; Steinmetz and Constantinidis, 1995) and, whilst something of a ‘poor relation’ to neighbouring area LIP, holds the advantage of accessibility to sub-pial electrodes. Richter et al. (2017) confirm that beta GC is greater in the 7A-to-V1 than V1-to-7A direction, and enhanced by spatial attention to the site of the V1 electrode in question. Furthermore, they show that forward gamma GC to area V4 from the same V1 electrode is then enhanced: specifically, that there is epoch-by-epoch correlation in the joint magnitudes of 7A-to-V1 beta GC and V1-to-V4 gamma GC, with the effect of the former upon the latter being delayed by about 100 msec. This influence of 7A over V1 must be mediated indirectly, via V2 and/or other prestriate areas, as direct afferents from 7A to V1 are rather sparse, and limited to peripheral field within the calcarine sulcus, well away from the recording sites upon the occipital surface of V1 (Borra and Rockland, 2011). Hence, transmission from 7A to V1 is best interpreted as a cascaded, high-level precision signal, predicting the behavioural significance of a particular visual locus; given the known physiology of area 7A, it is unlikely to be predicting featural content, and the observed influence was not suppressive. The outcome of the study can thus be interpreted as an example of FB precision acting to enhance the gain of FF error transmission.

## 4 Predictive physiology

Aligning with the traditional view of ‘percepts as hypotheses’ (Gregory, 1980), ‘predictions’ under gPC are essentially descriptions of what is being viewed, according to the current interpretation of retinal image content. Representations grow progressively more abstract and spatially invariant at higher levels, each level attempting to predict details of the less abstract representation present at a subordinate level. This is as true in the temporal dimension as in the spatial dimension – higher levels employ longer windows of temporal integration, as evidenced by varied lines of investigation in humans (Hasson et al., 2008; Gauthier et al., 2012; Honey et al., 2012; Tang et al., 2014), monkeys (Murray et al., 2014; Chaudhuri et al., 2015; Cocchi et al., 2016) and rodents (Piasini et al., 2021; Siegle et al., 2021). Thus predictions do encompass temporal forecasts, and this indeed is the approach adopted by most experimental paradigms for manipulating subjects’ state of expectation: once accustomed to a certain series of events, event (i) predicts the occurrence of event (i + 1). Intuitively, such procedures might be most effective when the sequence is non-arbitrary and ‘natural’ – such as dynamic facial expressions, gestures, locomotor movements, or object trajectories obeying Newtonian laws of gravity and motion. Although applied in non-invasive human studies (Alink et al., 2010; Kok et al., 2013; van Pelt et al., 2016; Hogendoorn and Burkitt, 2018; Thomas et al., 2018; Blom et al., 2020; Todorova et al., 2021) direct analogues in the realm of monkey neurophysiology are absent; instead, the common strategy has been to employ arbitrary sequences of static images, with the intention of generating various forms of ‘statistical’ expectation.

### 4.1 Statistical expectation and error encoding

Statistical learning is a recognised and well-studied phenomenon in humans, referring to automatic memorisation of temporal regularities in routine experience, even if the events in question lack much behavioural relevance or escape explicit attention. Various procedures exploiting this form of statistical expectation have sought to determine neural correlates in IT cortex whilst monkeys passively view streams of complex objects. The regularity of the stimulus paradigm enables an accurate temporal forecast of what is to be presented next, where, and when – although ‘what’ is typically the experimental variable, ‘where’ and ‘when’ remaining consistent from trial to trial. By their nature, statistical predictions should be instantiated prior to stimulus onset: thus their neural mechanisms might be isolated during a prestimulus period, and affects upon neural activity observable immediately from response onset.

The requisite procedure is to ‘train’ (i.e., simply expose) monkey subjects to repeated identical short sequences of highly distinctive stimuli, normally metrically standardised colour images of objects and animals familiar to humans (but likely merely abstract forms from the monkeys’ perspective) – typically thousands of times over several weeks. Upon subsequent testing, a few trials with altered sequences are interspersed amongst the regular ones; the common finding has been that the unpredictable ‘deviant’ items in these sequences elicit significantly larger responses, consistent with an interpretation that the lesser responses to standard items reflect routine predictive suppression (Meyer and Olson, 2011; Meyer et al., 2014; Ramachandran et al., 2016, 2017; Kaposvari et al., 2018; Esmailpour et al., 2022). In the first study of this nature Meyer and Olson (2011) found 41% (33/81) of neurons in area TE of IT cortex (with unknown laminar location) displaying this property at a statistical threshold of *p* < 0.05. Neural dynamics (averaged from this subset of 33 putative ERR units) showed that the deviant responses were marginally delayed, by 6 ms, with respect to standard responses, and that the enhanced activity was present from the outset of the response. Or, in other words, that the state of expectation slightly accelerated the response to a predicted stimulus, and reduced its magnitude from the moment of onset.

Subsequent work has clarified that, essentially, each stimulus in a learnt sequence predicts its immediate successor, and that there are only minor indications of longer range, so called ‘non-adjacent dependencies’ (Meyer et al., 2014; Kaposvari et al., 2018). Taking advantage of this, Esmailpour et al. (2022) used learnt stimulus triplets, exchanging the second item between triplets in a minority of trials during testing. Hence, in these deviant triplets, both the second and third items should elicit an error response, as the third item is no longer predicted by its immediate predecessor. Testing was conducted in interconnected sites of IT and ventrolateral prefrontal cortex, the latter a part of frontal area 45 identified by prior fMRI mapping and the former in a region of ventral superior temporal sulcal cortex reactive to electrical stimulation of area 45. As anticipated, both the 2nd and 3rd items of deviant triplets elicited enhanced, error-like signals; these were registered in both aggregate ‘multiunit’ neural spiking and LFP, recorded in both IT and area 45 cortex in a non-laminar resolved fashion. Error signals, obtained as such by subtracting standard and deviant responses to the same stimulus items occurred at longer latency in area 45 (measured as a 44–88 ms delay for multiunit activity), consistent with FF transmission from IT. For both areas of cortex, deviant responses entailed enhanced gamma LFP, in line with evidence reviewed above linking gamma to error signalling. In addition, deviance reduced beta power in LFP signals, again in both areas; the report also notes that, in standard triplets, beta LFP was greater for the 1st and 2nd items, that both predict a successor, in comparison to the 3rd, that does not. The reduction of beta activity occasioned by deviant items perhaps signifies that the unexpected stimulus acts to erode predictive representations of the expected stimulus at higher levels: both in area 45 with backward output to IT, and in IT with backward output to prestriate cortex.

In all these experiments, a correct prediction is compared to a misprediction. Logically, each might be compared to a neutral condition, i.e., the absence of any prediction. Two studies addressing this issue, as to whether error signals principally comprise predictive suppression, or ‘surprise enhancement’ arrived at different conclusions: one favouring predictive suppression as the dominant component (Ramachandran et al., 2017), the other surprise enhancement (Kaposvari et al., 2018). It is possible that technical details of stimulus presentation such as image and sequence duration, and the presence or absence of an interstimulus interval are confounding factors here. However, it has been explicitly questioned whether surprise enhancement can properly substitute for predictive suppression within the conceptual framework of predictive coding (Feuerriegel et al., 2021). This issue requires greater consideration of the neural mechanism(s) generating an error response, and will be returned to in discussion.

None of the above studies considers error signalling in relation to different classes of stimulus selectivity – indeed IT responses to objects are notoriously difficult to classify in systematic fashion. Face sensitive neurons, however, show selectivity for head orientation, such as frontal or profile view, and for individually specific configurations of facial features – often characterised as ‘view’ and ‘identity’ selectivity – and offering a basis for the investigation of selective error signalling. The statistical learning study in question here (Schwiedrzik and Freiwald, 2017) focused upon ‘ML’, a mid-order face patch known to display view selectivity. Subsequent face patches ‘AL’ and, at the highest level, ‘AM’ progressively develop identity selectivity accompanied by view invariance (Freiwald and Tsao, 2010). This raises an intriguing question: what is the nature of error signalling in a face patch, ML encoding view yet likely receiving backward predictions from areas in which view selectivity is eroded in favour of identity coding?

Following standard practice Schwiedrzik and Freiwald (2017) allowed monkeys an extensive period (30 days) to learn nine fixed pairs of ‘predictor’ and ‘successor’ achromatic human faces; subsequent testing introduced deviant pairings that were designed to elicit errors in expected view, or expected identity, or both view and identity. The nine learnt pairs comprised all possible combinations of frontal, left profile and right profile view, all 18 faces having a unique identity. A consequent flaw in this design is that any unfamiliar pairing of these 18 face images must necessarily evoke an identity error; thus, to achieve a view-only error condition, it was necessary to employ an unfamiliar (i.e., untrained) view (e.g., reversing a left profile to bcome a right profile of the same identity). As this unfamiliar view was presented as a predictor face, it is uncertain what expectation it should give rise to. And, its use as the predictor face betrays a more fundamental problem with the design. Unaccountably, the authors did not classify the error conditions in respect of the departure of the deviant successor stimulus from the expected stimulus, but by the reverse: the departure of the predictor stimulus of a deviant pair from the familiar predictor stimulus preceding that successor. In consequence, although the study was successful in identfying as many as 64% of ML neurons expressing some form of prediction error (an enhanced response to the successor stimulus in the context of an unfamiliar pairing) the classification of these error signals must be amended.

**Figure 4:**
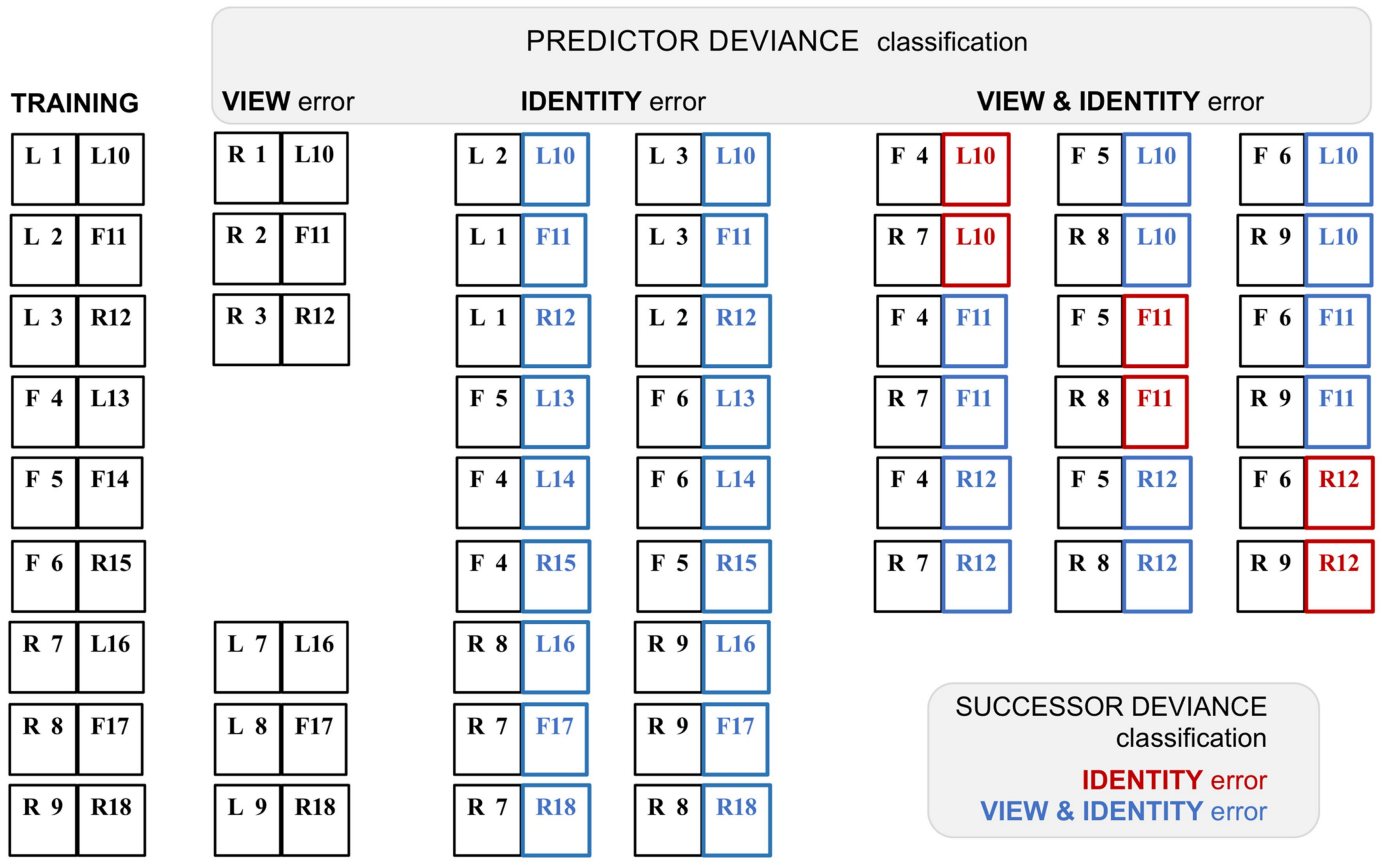
Variant classification of stimulus pairings by predictor and successor deviance. A schematic rendition of the sequential stimulus pairings employed in the study by Schwiedrzik and Freiwald (2017). Images were greyscale human faces of either sex (with bare scalp) shown in frontal (F) or 60° left (L) or right (R) profile viewpoint. There were nine training pairs, showing faces from eighteen different individuals, tagged 1–18. In subsequent testing allied to neurophysiological recording, a proportion of trials presented faces that were re-coupled so as to deviate from the trained pairings; faces 1–9 and 10–18 retained their position as first and second stimuli in the sequence respectively, i.e., serving as ‘predictor’ or ‘successor’ images. The deviant pairings were configured to confound expectation with respect to face viewpoint, or face identity, or both viewpoint and identity. However, such a classification can be contingent on the deviation of either the predictor, or the successor, from the trained pairing: the study employed the former (‘predictor deviance’), whereas only the latter (‘successor deviance’) is accurate for assessing prediction error (since only the second sequence in a pair may honour or breach expectation). Training pairs are shown at left, followed by columnar arrangement of the three sets of deviant test pairings as classified by predictor deviance. Re-classification by successor deviance is indicated by red (‘identity’) or blue (‘view & identity’) colour coding of the second stimulus in each pair. Note that the report does not specify precisely which recoupled pairs were presented experimentally; the deviant pairings shown here are the logically permissible permutations. The six view-error pairings were created by left–right (or right–left) reversal of the six profile-view predictor stimuli, thus preserving the sequence of face identity, at the expense of presenting a non-trained face image of (possibly) limited predictive status. Exhaustive recoupling of the 18 trained face images generates 72 fresh pairings, comprising 18 identity-error pairs and 54 view & identity-error pairs (as classified by predictor deviance). All 18 identity-error pairs reclassify to view & identity under successor deviance. Of the 54 view & identity-error pairs one third reclassify to identity-error under successor deviance. The figure shows just 18 of these pairings (corresponding to the first triplet of trained successor stimuli) to illustrate how this proportion arises; the second and third triplets of trained successor stimuli follow a similar pattern.

That task is achievable; the deviant pairings retained predictor and successor status of the trained stimuli, and hence the composition of these pairings can be inferred (see Figure 4). Following the authors’ criteria (‘predictor deviance’), each successor face permits two deviant pairings classified as identity only (‘ID’), and six classified as view and identity (‘V&I’). Reclassifying by conventional ‘successor deviance’ (denoted by appending an *), the two ID pairs become *V&I, and two of the six V&I pairs reclassify to *ID, whilst four persist as *V&I. The study identified systematic variants in the timecourse of error signalling: whilst view-error signals were comparatively weaker and short-lived, both ID and V&I trials showed more prolonged error signalling, enduring from peak response (at 116–125 ms) to around 400–500 ms post stimulus onset. Tellingly, the response profiles of the ID and V&I error signals (as classified by predictor deviance) are highly similar, both comprising mainly *V&I error signals as reclassified by successor deviance. Although the report confirmed that ML neurons are inherently tuned to facial viewpoint, it inferred that their error signalling was dominated by identity selectivity inherited from descending predictive signals. This conclusion has two flaws. First (as noted above) the supposed ‘view-error’ trials were likely compromised by a failure of the unfamiliar predictor used in this condition to generate anymuch expectation. Second, the two other conditions both had a significant content of view prediction error. In fact the ID trials, with 100% view-error content as reclassified to *V&I, display a marginally more robust average error signal than the V&I trials, with lesser (67%) view-error content if all potential deviant repairings (four *V&I and two *ID) were presented with equal frequency.

Such a revised interpretation of this study (Schwiedrzik and Freiwald, 2017) suggests that view-error is indeed the principal component of FF transmission from ML. This conclusion is also dictated by algorithmic formulations of predictive coding, which specify that predictive signals routed to ERR units should match their featural competence; they must do so, in order to allow a valid subtraction of one from the other. Admittedly it leaves unresolved the initial quandary, how view specific predictions might be generated by higher stations with laxer forms of view coding.

### 4.2 Probabilistic expectation and reported absence of error encoding

An alternative and more practical means to induce expectation, without resorting to an extensive learning period, is to simply repeat the same stimulus over and over again. However, any predictive suppression caused by so simplistic a procedure runs the risk of being confounded by a concurrent ‘neural fatigue’ effect, thought to be the consequence of cumulative synaptic depression along the FF pathway leading to the brain site of monitored activity; this is frequently referred to as ‘repetition suppression’, in distinction to predictive or expectation suppression (Vogels, 2016; Feuerriegel et al., 2021). Studies aiming to sidestep this problem have reported an absence of error signalling; three are reviewed below with a critical focus upon the nature of expectation delivered by the various paradigms.

One method to dissociate repetition and predictive suppression is as follows. Each trial presents a regular sequence of two stimuli (S1 and S2); S2 is either a repeat of S1 (‘*rep*’ trial), or an alternative stimulus (‘*alt*’ trial); trials are presented in *Rep* or *Alt* blocks, in which either *rep* trials or *alt* trials are much more frequent; finally all stimuli are unique, in that no stimulus is presented in more than one trial across the entire experimental session. The experimental logic here is that repetition suppression should occur (in *rep* trials) in both *Rep* and *Alt* blocks to an equal degree, whereas predictive suppression should be favoured by *Rep* blocks and absent (or much diminished) in *Alt* blocks. Hence in this design statistical regularities are learnt over a far shorter time course, within and across stimulus blocks lasting several minutes. It has been used to good effect in several human fMRI studies yielding results interpretable as predictive suppression, e.g., as initially demonstrated for the fusiform face area (Summerfield et al., 2008) – but this was not matched by attempts at replication in single unit-studies of monkey IT cortex (Kaliukhovich and Vogels, 2011; Vinken et al., 2018). These studies demonstrated robust repetition suppression (i.e., attenuated S2 responses in *rep* trials), but no more so in *Rep* blocks than *Alt* blocks, as might have been attributable to predictive suppression. Some factors underlying the negative outcome may be adduced as follows.

Firstly, both studies use population data of single or multi-unit activity, plus LFP signals to report variations in S2 response magnitude across trial type and block (normalised with respect to S1 response magnitude, which itself shows little variation). Thus any ERR unt activity might potentially be masked within the aggregate stimulus response – although other studies reviewed above demonstrate that this is not invariably the case, e.g., with regard to LFP signals at gamma frequencies. The concern lies more in the nature of the paradigm, particularly as applied to monkeys. Kaliukhovich and Vogels (2011) deployed it as a passive task, requiring no attention. They used two distinct stimulus sets, one comprised of gaussian-windowed natural stimuli (landscapes/animals/bodies/man-made artefacts/buildings), the other fractal patterns (complex forms with multiple geometric elements and textures). Such stimuli are good for evoking responses from IT cortex, but it is not clear that momentary presentation of a novel, abstract, behaviourally irrelevant stimulus will elicit much visual cognition. Logically, in order to be able to expect to see the same thing again (as ‘S2’), there must be a representation of that thing, some conception of it, that can be recalled from memory (and not just iconic memory). Here, as we might infer, no such cognition arose within a higher centre with backward, predictive output to the recorded region of IT cortex. This might well occur in a human subject looking at a face, or the Eifel Tower, but less so for a monkey seeing the latter, or a fractal.

**Figure 5:**
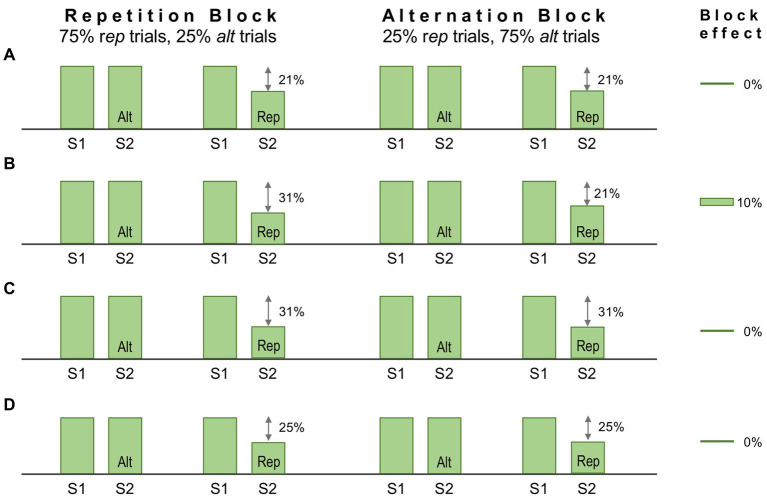
Schematic of the design, and some notional outcomes, of a repetition probability experiment. In this design stimuli are delivered sequentially, one pair (S1 and S2) per trial. The probability that S2 is a repeat of S1, or an alternative stimulus, is fixed within a block but varied between blocks, defining ‘repetition’ and ‘alternation’ blocks as indicated. Repetition suppression should be operative in all repetition (*rep*) trials irrespective of block. The statistical design should encourage expectation of a repeated stimulus in a repetition block, meaning that *rep* trials (but not *alt* trials) might also demonstrate predictive suppression. Conversely, in alternation blocks, repetition is not expected, or is less expected. Predictive suppression is therefore inferred by the presence of a block effect, i.e., different levels of response suppression in *rep* trials between the two blocks. **(A)** Single unit data for one subject reported by Vinken et al. (2018). Recordings were made in face patch ML to synthetic human face images depicting frontal views of distinctive individuals. Both blocks showed the same decrement of response, 21% in *rep* trials. Hence there is a zero block effect (0%). **(B)** Notional data for recordings from a face patch with sensitivity to face identity. Predictive suppression allied to repetition suppression causes a larger 31% response decrement in *rep* trials in the repetition block. In the alternation block *rep* trial responses retain a 21% decrement, leading to a block effect of 10%. **(C)** Notional data for face patch ML (possessing face view but not identity sensitivity) invoking the assumption that a frontal view of a face (irrespective of identity) is equally predictable for *rep* trials in both blocks. Thus the combined effect of repetition and predictive suppression is equal in both blocks, and the block effect is 0%. **(D)** Notional data for face patch ML invoking the broader (and possibly more valid) assumption that a frontal view of a face is equally predictable for *all* S1 and *all* S2 stimuli irrespective of trial or block type (since all stimuli are such). In consequence all responses show an equal response decrement due to predictive suppression (although there is no standard response against which to measure it). As repetition suppression continues to operate equally in in *rep* trials in both blocks, the block effect is again 0%.

Mindful of these limitations, Vinken et al. (2018) modified the procedure to involve an attentive task discriminating face stimuli – i.e. to report whether S1 and S2 were the same or different faces. Performance on this task demonstrated that the monkeys were indeed sensitive to repetition probability, as their decisions were biased toward ‘same’ reports in *Rep* blocks, and toward ‘different’ reports in *Alt* blocks. Recordings were obtained from face patch ML identified by prior fMRI mapping. Despite these modifications the outcome was much the same as before: repetition suppression could be detected in averaged single and multi-unit data, and in high gamma LFP signals, but there was no evidence for predictive suppression, as apparent from the absence of any block effect. Figure 5A summarises the single unit data for one subject showing a 21% reduction in activity in *rep* trials equal in both blocks. The stimuli used in this study were synthetic human faces, realistic depictions of highly distinctive individuals but – and here is the likely problem – all presenting a uniform frontal view. Face patch ML, as noted above, is view selective but expresses little or no selectivity for face identity (Freiwald and Tsao, 2010). The experiment would have a greater likelihood of success if recordings were obtained from a higher face patch (e.g., AM) with featural competence to match the same/different predictions of face identity (e.g., as indicated in Figure 5B). For ML, the *Rep* and *Alt* blocks are essentially equivalent, as they both allow prediction of S2 as a frontal face view (Figure 5C). Now, following this line of reasoning, it might be argued that the same should be true of *alt* trials, given that they present the same, 100% probability that S2 will be a frontal face. However, there is an extra twist here: the design of the experiment permits equally certain prediction of S1, given that it too is always a frontal face (and regularly timed from fixation onset). Hence all stimuli, across all trial and block types, are equally predictable as frontal face views, and equally likely to undergo predictive suppression – leaving repetition suppression, as effected in S2 of *rep* trials, as the only mechanism operating differentially upon neural activity in ML (Figure 5D). Qualitatively, therefore, the response patterns depicted in Figures 5A,C,D are indistinguishable, and the magnitude of response deficit observed in *rep* trials allows no inference of the relative severity of repetition and predictive suppression.

A contrasting means of regulating expectation is by means of the ‘oddball’ paradigm, as frequently used in auditory studies (Chao et al., 2018). This has been adapted for study of areas V1 and V4 with sequences of orthogonal gratings, ‘A’ and ‘B’, where a single instance of B interrupts repetition of A, such as AAABA (Solomon et al., 2021). Successive responses to A are of reduced magnitude, attributable to either or both forms of suppression, but the key strategy here is to compare responses to B in trials when it appears at a deviant 3rd or 5th position in the sequence (10% each) compared to a regular 4th position (80%). Enhanced responses to deviant B stimuli would indicate error signalling, as a smaller response to regular B stimuli could only be attributable to predictive suppression, contingent upon sequence learning. However no such difference was observed; and despite extending exposure to the regular sequence before testing deviant sequences, plus exploring factors such as grating size, or presentation time, the authors ultimately concluded that spiking and LFP activity elicited in the superficial layers, of both areas, by deviant and regular B stimuli was “near identical” (Solomon et al., 2021). The pertinent question here is whether this paradigm, deployed as a passive viewing task, did actually induce states of expectation as intended. Even human subjects, with greater implicit cognition of an ‘oddball’ stimulus, only displayed pattern violation EEG activity when explicitly tasked to detect oddballs (Solomon et al., 2021). That monkeys should develop expectation contingent upon position-dependent cognition of an unattended stimulus-sequence is far from given. Lacking that, default expectation (A) = expectation (B) = 50% for every grating seen (since A and B reversed role as oddball in alternate sessions). It is possible, perhaps, that statistical learning of transitional probabilities (as described in 4.1) developed across the course of multiple sessions; if so, this would simply reinforce the default 50% expectation since, during the course of the experiment, mid-sequence pairings AA, AB, BB and BA were presented equally frequently. Assessing the paradigm from this perspective, absent evidence of error encoding poses little challenge to PC theory.

### 4.3 Separate encoding of prediction and error

This section summarises a couple of studies not merely reporting neural signalling of prediction error, but dissociating this from signals encoding predictions themselves (Bell et al., 2016; Bastos et al., 2020). Both happen to manipulate expectation by a relatively simple means, maintaining a fixed frequency of presentation of a certain stimulus item within blocks ranging from 0 to 100% between blocks. Bell et al. (2016) examined comparative responses to greyscale images of monkey faces, and various types of fruit. Recordings (not laminar-resolved) in areas TEO and TE of IT cortex included, but were not restricted to the local network of face sensitive modules (i.e., face patch ML, etc). A majority of responsive neurons (61%) had significantly higher responses to the face stimuli; virtually none (1%) preferred the fruit stimuli (that, as mooted above, might appear as unrecognised, non-face abstract forms). These two stimulus types were presented as cues in the context of a match-to sample task; to make the task more demanding of attention, the images were degraded by the addition of a low, or high level of Gaussian noise. Performance on the task indicated that, as with Vinken et al. (2018), the subjects were highly sensitive to the blocked variations in probability (0, 25, 50, 75 & 100%), biasing their choices of fruit versus face accordingly, and accentuated by the greater stimulus uncertainty imposed by the high-noise condition. Furthermore the performance data enabled theoretical modelling of trial-by-trial fluctuations in the subject’s state of expectation, the variable *p(face)*.

**Figure 6:**
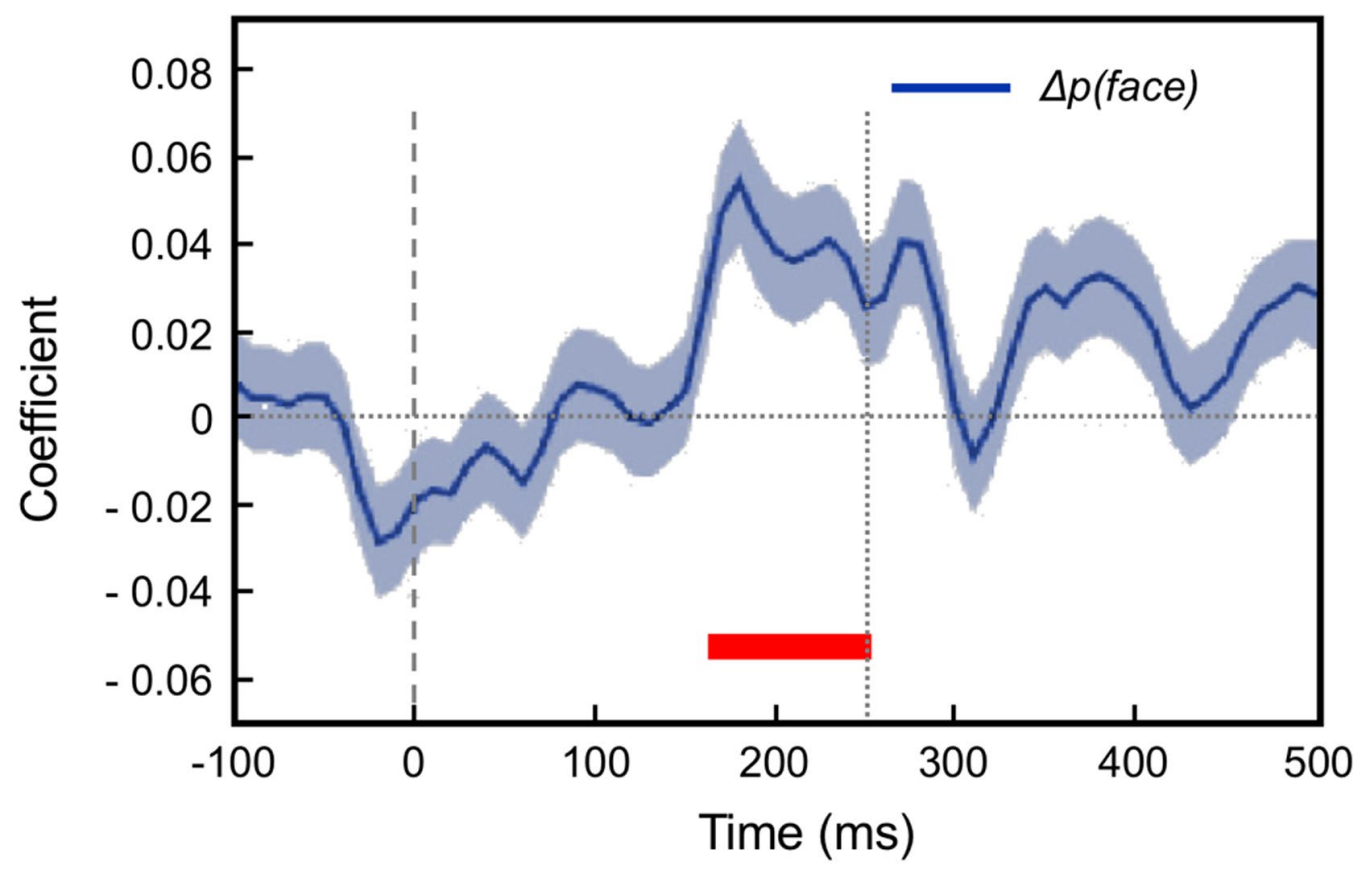
Population encoding of prediction error determined for IT cortex (Bell et al., 2016, 2017). The chart shows the timecourse of the regression coefficient for the factor *Δp(face)*, a measure of prediction error, as determined from the population response of neurons in inferotemporal cortex to a monkey face stimulus in the low noise condition of this study. The regression analysis was compiled from response data gathered across stimulus blocks controlling face expectation by varying the block frequency of presentation of either a face (or fruit) stimulus from 0 to (25-50-75) to 100%. The factor *Δp(face)* was most prominent in trials in response to a face stimulus when a face was most unexpected. The figure shown here is reproduced from post-publication correspondence (Bell et al., 2017; Vinken and Vogels, 2017) concerning a notional role of repetition suppression (i.e., biophysical fatigue) in promoting a reduced response to an expected face stimulus, that would otherwise be attributed to predictive suppression; it is a modified version of regression modelling with added precautions in that regard. The red bar indicates the time period where the coefficient is significant, from approximately 150–250 ms. Notably, that timecourse is not consistent with repetition suppression, as relief from neural fatigue in a stimulus block with low face expectation should begin to enhance activity earlier, immediately upon response onset at approximately 100 ms. Reproduced by permission of Elsevier.

The modelling procedure used for this purpose derives from reinforcement leaning theory (Sutton and Barto, 1998) and additionally provides a measure of trial-by-trial ‘surprise’, *Δp(face)*, that serves as an index of prediction error. The authors then performed multivariate regression analysis of each recorded neuron’s spiking rate across trials using factors of stimulus identity (face or fruit), *p(face)* and *Δp(face)* and determined how the population-average regression coefficient for each factor varied across the time course of the trial. This demonstrated global encoding of *Δp(face)*/prediction error (see Figure 6), and also stimulus identity, but not *p(face).* Notably, all recorded neurons (including those deemed visually unresponsive) were submitted to this analysis, and whereas a large proportion of neurons emerged with near-zero coefficients on all factors, small minorities responded either positively, or negatively to each factor – but uniquely, were equally balanced in respect to *p(face),* so yielding the null global encoding of this factor. Two further significant observations followed: firstly, this (bipolar) encoding of *p(face)* was present in the prestimulus period and persisted throughout the trial, unlike the other two factors whose earliest influence upon neural activity initiated 100 msec or so after stimulus onset; secondly, cross-factor correlation analysis revealed orthogonal encoding of *p(face)* and *Δp(face)* across the population, i.e., a dissociation consistent with separate EXP and ERR units, whereas both factors (as anticipated) were associated with a high face/fruit preference in favour of faces.

If the work of Bell et al. (2016, 2017) remains, to date, the foremost explicit identification of single-unit encoding of stimulus predictions (at least in monkey visual cortex) it is nicely complemented by the study of Bastos et al. (2020) focusing upon spectral analysis of gross population activity, MUA and LFP, but of known superficial (1–4) versus deep (5–6) layer of origin. This multilaminar activity is recorded simultaneously by multiple-contact electrodes carefully positioned to span all layers when introduced perpendicular to sub-dural cortical surface – as proved practical with regard to prestriate area V4, parietal area 7A, and a region of posterolateral prefrontal cortex (PFC). Non-laminar resolved recordings were also made from areas LIP and FEF, but of all these areas only V4 has the high level of visual feature selectivity commensurate with the match-to-sample task that was used to manipulate stimulus expectation. The cue stimulus, presented centrally, was followed by a choice array of three items presented eccentrically: two foils, plus one matching the cue. Crucially, trials were delivered in blocks which either maintained one stimulus as cue throughout the block (‘100%’ condition) or presented all three as cue with equal frequency (‘33%’ condition). These stimuli were natural objects of bright, near uniform colouration, and thus easily memorised and distinctive, if merely abstract images from the perspective of the monkey subjects. Neural activity recorded during cue presentation revealed clear stimulus preferences amongst different V4 sites. The preferred cue at a given site was found to elicit maximal MUA and gamma LFP signals, from the superficial layers, when presented in the 33% (‘unpredicted’) condition; conversely, it elicited maximal beta and alpha LFP signals, from the deep layers, when presented in the 100% (‘predicted’) condition. The former finding aligns with previous work linking superficial gamma activity in V4 to FF error signalling, in that the larger gamma response plausibly reflects a lower level of predictive suppression enacted within the 33% condition. Notably, the study also reports a finding consonant with that of Richter et al. (2017) as described above, in that there was a significant trial-by-trial correlation between beta LFP power recorded in area 7A, and gamma LFP power expressed by V4; as before, this is interpretable as a top-down precision effect, although whether mediated directly upon V4, and/or indirectly via V1 cannot be ascertained.

The low frequency (alpha/beta) LFP activity recorded in the deep layers of V4 acted like a proxy measure for backward predictive signals. The alpha/beta LFP power differential between deep and superficial layers was most marked when a 100% expected cue matched a V4 site’s preferred stimulus, and this was true not only following the cue stimulus onset (as stated above), but also during the pre-stimulus period. In other words, similar to IT cortex in the study of Bell et al. (2016), V4 appeared to register stimulus-specific predictive signals in the regularly timed 1 s period between fixation and cue stimulus onset. Finally, simultaneous recording from several areas enabled a search for the higher-level source of predictive signals, as earmarked by area-to-area Granger causality in the alpha/beta frequency range. The deep layers of PFC were found to be strongest candidate for this role during the pre-stimulus period, later abetted by the superficial layers of the same area following onset of the cue stimulus.

A final comment is merited on the nature of adaptation. ‘Neural fatigue’ is a legacy term for (usefully) adaptive regulation of synaptic efficacy, acting to calibrate neural sensitivity within a changeable sensory environment (Thompson and Burr, 2009) – what might be classed as perceptual learning as opposed to perceptual inference. Under gPC theory, both can be considered mechanisms for reducing prediction error (Auksztulewicz and Friston, 2016): perceptual learning plays out over multiple trials, theoretically encompassing processes such as resetting priors and adjusting the parameters governing (predictions of) precision; its panoply of neural mechanisms include synaptic plasticity. Perceptual inference operates over the course of peristimulus time in a single trial. The evidence presented here (Sections 4 & 5) reporting average neural responses over repeated trials, uniformly relates to perceptual inference. Concurrent adaptive changes may be observed but not commented in detail; e,g, Bastos et al. (2020) simply note that repetition effects developed over dozens of trials, correlating with the time course of improved behavioural performance in their task. From this perspective, paradigms designed to distinguish predictive suppression from repetition suppression are effectively isolating steady-state error signalling (perceptual inference) from longer-term adaptive modification of error signalling (perceptual learning).

### 4.4 Natural expectation

Everyday experience accumulates innumerable priors for interpreting natural scenes – what might be termed a knowledge of ‘visual ecology’ (Gibson, 1979). For example, scenic context and regular positional relationships can facilitate object recognition (Kaiser et al., 2019), whilst familiarity with natural lighting informs basic percepts of object shape, surface colour and glossiness (Murray and Adams, 2019). The light-from-above prior (Adams et al., 2004) can determine if a shaded, curved surface appears concave or convex, but may itself be trumped by other priors: e.g. the face-mask illusion, that is contingent on our knowledge that human heads are invariably convex (Gregory, 1980). Heuristically, ‘we see what we expect to see’ (de Lange et al., 2018); in gPC terms backward-going predictions mediate the effect of priors by adjusting the balance of activity between EXP and ERR units at subordinate levels. There is, in other words, a rich vein of perceptual phenomena ripe for neurophysiological exploitation. The following examples span the visual hierarchy, with the common thread that the ‘predictions’ here are essentially stimulus evoked interpretations – hypotheses – of what is currently being seen, as opposed to statistically sourced temporal expectations.

**Figure 7:**
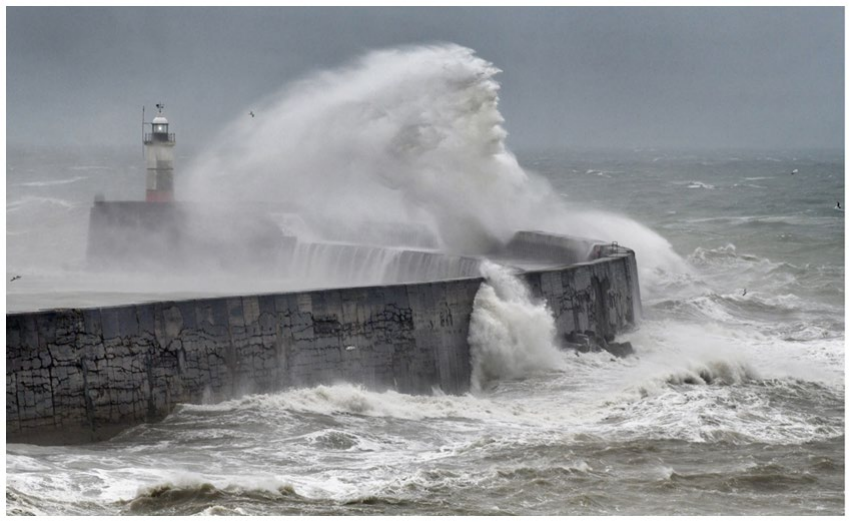
Pareidolia – a face in the waves. This striking image was captured during a storm at Newhaven harbour, United kingdom. Fittingly, the ‘face’ evokes the ancient Greek god of the sea, Poseidon. With permission Jeff Overs.

The human brain has a well-known readiness for seeing faces where none truly exist (‘pareidolia’: see Figure 7 for an example) – a kind of default expectation. It is hard to ascertain if the same is true of the monkey (Taubert et al., 2022), but the hierarchical organisation of the face system in IT cortex is similar in the two species, consisting of six or so discrete patches where neurons respond specifically to face-like patterns (Grimaldi et al., 2016; Hesse and Tsao, 2020). Simultaneous recording from face patches at posterior, central and anterior levels of IT cortex (pIT, cIT and aIT) found a subset of cells with responses consistent with ERR units (Issa et al., 2018). The stimulus set comprised synthetic images of macaque faces with either a typical, or atypical (i.e., distorted) configuration of face parts, such as eyes and snout. All were vetted to ensure approximate equality in their ability to activate neurons in the lowest-level face patch (i.e., pIT), known to respond particularly to eye-like images embodied within a face-like outline (Issa and DiCarlo, 2012). Face neurons at the apex of the hierarchy (aIT) maintained a consistent preference for typical faces, in contrast to the earlier levels pIT and cIT, where some neurons responded better to atypical faces. This preference, however, only began to develop about 30 msec after neural response onset (as measured by comparing cumulative spikes within windows 60-90 ms and 100-130 ms post stimulus onset). Simultaneous recording allowed close comparison of neural dynamics across the three hierarchical levels and indicated causation, in that, across images, late phase activity in pIT was negatively correlated with early phase activity in both cIT and aIT. This is what is expected if a backward predictive (i.e., descriptive) signal of a properly configured face acts to suppress activity within a subpopulation of face-sensitive ERR units at a lower level. Advantageously, the study reports the responses of neurons at an individual level (as opposed to a single population metric for each area), so providing estimates of 37, 30 and 0% for neurons with error-like responses in pIT, cIT and aIT, respectively. Smaller fractions (7.0, 8.6 & 2.5%) showed the reverse behaviour (i.e., a significant increment in typical vs. atypical stimulus preference between the early and late response phases). The absence of ERR units from what is considered to be the apex of a hierarchical chain (aIT) makes obvious sense, although the implications for the intrinsic architecture of such an area have yet to be explored. As the recordings were not laminar resolved, there is no evidence for a majority supragranular location of error responses. However, as the authors remark, it is clearly significant to be able to distinguish putative error and non-error (or ‘state estimating’) classes of neurons, as only the latter ‘are truly reflective of the tuning preferences of that IT processing stage’ (Issa et al., 2018).

**Figure 8:**
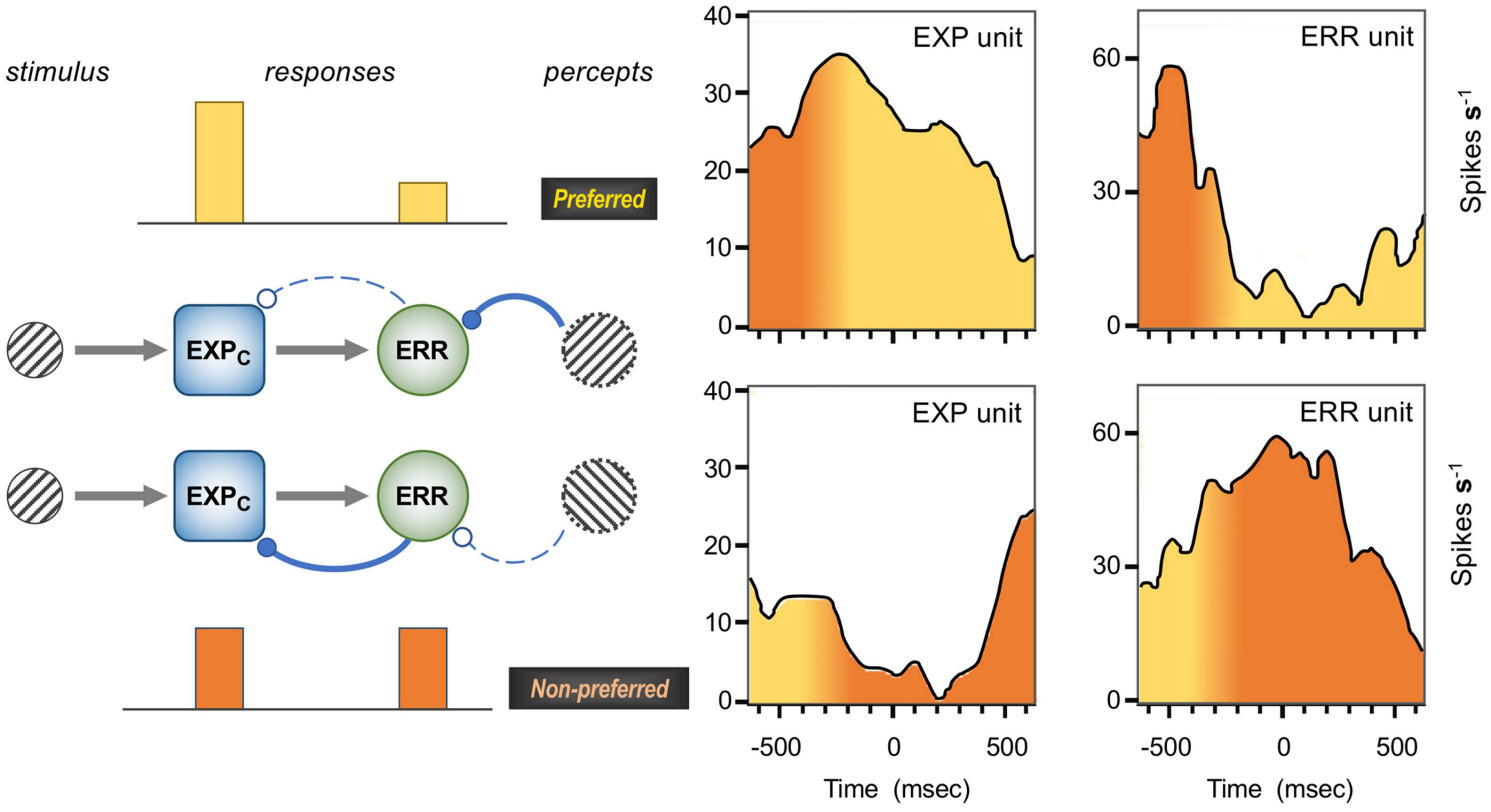
Inferred EXP unit and ERR unit activity during binocular rivalry. Right: the average activity of two orientation selective cells in area V4 during dichoptic (rivalrous) viewing of orthogonal gratings (Leopold and Logothetis, 1996), here interpreted as an EXP unit and an ERR unit. The timescale shows a period of 650 ms before and after the behaviourally trained monkey’s report of a change in percept; multiple transitions from the non-preferred to preferred orientation (top row), and from the preferred to non-preferred orientation (lower row) are registered at time zero, the instant of perceptual report. Reports were made manually, and although they must follow the instant of perceptual reversal with a certain latency, the precise timing is unknowable. The yellow-to-orange (preferred-to-nonpreferred) shading shown here is configured for correspondence to the model at left. The key observation is that the EXP unit is more active while the percept matches its preferred orientation, and the ERR unit shows the reverse behaviour. Left: a schematic model of the interactions between EXP_C_ and ERR units sharing similar orientation selectivity during alternating phases of the two percepts. The feedforward drive to the EXP unit is constant and in turn activates a negative feedback loop with the ERR unit. When the percept is concordant with the neuron’s preferred orientation, the ERR unit undergoes predictive suppression from a higher level in the hierarchy, which in turn disinhibits the EXP unit. Conversely, when the percept is discordant with the neurons’ preference and there is no predictive suppression, the two units achieve a more balanced level of activation. The greater FF output of the ERR unit in this state is thought to exert a cumulative destabilising effect at higher levels, acting to promote the next perceptual reversal (Hohwy et al., 2008). Grey arrows: FF excitation; blue, solid/dashed lines with filled/open ball-head contacts represent active/inactive inhibitory transmission. Activity profiles reproduced with permission SpingerNature.

Bistable percepts provide a means of examining the neural basis of shifts in visual cognition at higher levels, whilst the retinal image remains unchanged. Binocular rivalry provides the additional advantage of allowing arbitrary (dichoptic) retinal image content, determined by the featural competence of the cortical area under study: e.g. opposite directions of motion for study of V5 (Logothetis and Schall, 1989), and orthogonal orientations for V4 (Leopold and Logothetis, 1996). Each of these studies reported a minority of neurons whose stimulus preference (respectively for direction and orientation) could be determined during non-rivalrous viewing, and whose activity subsequently correlated with the monkey’s alternating perceptual reports during rivalrous viewing. These fractions were 22% (13/59) for V5 and 24% (16/68) for V4. Importantly, in the current context (but an inexplicable-*cum*-inconsequential detail at that time) each of these samples comprised some neurons whose activity was enhanced whilst congruent with the perceptual report and some whose activity was, conversely, suppressed; the ratio of these two classes was about 1:1 for V5, and 2:1 for V4. Considered from the perspective of predictive coding theory, the former group acts like EXP units, encoding what is currently being perceived, whilst the latter unanticipated/aberrant suppressive neurons are natural candidates for ERR units (see Figure 8). In retrospect, the alternating percepts of the rivalry paradigm provide an ideal vehicle for distinguishing EXP and ERR functionality. Future studies exploiting this approach could usefully examine the laminar locations of these units, and analyse the interplay of perceptual and neural dynamics.

At a primary level, such as studied in area V1, contextual predictions reflect statistical properties of the natural environment, largely shorn of any cognitive referents – elemental features such as the spatial uniformity of texture and chromaticity, or the continuity of lines and edges. For example Rao and Ballard (1999) modelled error signalling as predictive suppression of local edge detectors by higher level representations of an extended contour, so generating the documented phenomenon of ‘end-stopping’ in V1. Subsequent investigation of such (so-termed) ‘iso-orientation surround suppression’ using grating stimuli confirms partial causation by FB cortical processes, inferred from the timecourse and laminar profile of such effects (Bair et al., 2003; Bijanzadeh et al., 2018) – which is not to equate all instances of surround suppression with predictive suppression, since the former is also observed in layers where FF neurons are sparse, or absent. Rather, the model of Rao and Ballard implies that the orientation tuning of surround suppression should be keenest in the superficial layers of V1 with peak density of FF neurons – as indeed observed (Bijanzadeh et al., 2018) – and that even there, the severity of surround suppression should show strong variation across presumptive ERR and non-ERR classes of neurons. Such variation has been confirmed, for natural images as well as regular grating stimuli (Coen-Cagli et al., 2015); and, notably, all three of the above-mentioned studies of macaque V1 were conducted under anaesthesia, indicating a level of automaticity on the part of contextual suppressive effects distinct from various forms of cognitive expectation recounted above.

Aiming to test this interpretation of surround suppression more rigorously, Uran et al. (2022) devised a metric of image predictability exploiting a pair of artificial neural networks. A first network was trained in a self-supervised learning protocol to generate image content for the (masked) central patch of a fivefold larger natural image. A second convolutional network, trained by supervised learning for object recognition (OR-CNN), was used to determine the similarity of the synthetic image content to the original: submission of the twin full images to the OR-CNN caused quantifiably different patterns of activity amongst those artificial neurons responsive to the central patch. This metric of image predictability is thus the inverse of the OR-CNN’s differential response to the synthetic image. Multiple natural images, so assessed, were used as stimuli for monkeys in a passive fixation task, each scaled to match the central patch to the RF size of multiunit activity recorded from superficial sites in area V1. The general finding was that V1 activity decreased with image predictability. The strongest relationship was between (low) late V1 firing rates (recorded 200–600 ms post stimulus onset) and (high) predictability assessed specifically from the deeper layers of the OR-CNN. Due to the progressive convolutional construction of CNNs, artificial neurons in deeper layers express higher level features, abstracted over larger image sub-regions – though the nature of these features typically defies simple description. The study is nonetheless supportive of the gPC thesis that higher level contextual predictions can exert suppressive effects upon ERR units in subordinate visual areas, albeit here detected only as a net decrease in the average, ‘multiunit’ sample of superficial layer activity.

A significant adjunct to this study is that LFP gamma activity in V1 was found to show the opposite behaviour, correlating positively with image predictability, particularly as indexed by the early layers of the OR-CNN (Uran et al., 2022). This implies susceptibility to lower-level features, and accords with previous reports that large regular stimuli (such as gratings, or patches of uniform chromaticity) elicit high gamma activity from area V1 (Ray and Maunsell, 2010; Jia et al., 2011; Peter et al., 2019; Gieselmann and Thiele, 2022). It demonstrates that gamma activity is not invariably a proxy of error signalling, as emerged in studies cited above (Bastos et al., 2020; Esmailpour et al., 2022) – that examined fluctuations in gamma activity occasioned by changes in cognitive states of visual expectation whilst viewing the identical stimulus. By contrast, increments in stimulus size (coupled with cognitive neutrality) can recruit coordinated responses from larger neural populations, facilitating local gamma synchronisation of LFP signals. Precisely how the neural mechanisms of the gamma cycle tie in to FF error signalling remains to be elucidated (Ferro et al., 2021).

## 5 Predictive coding in mouse V1

In counterpoint to the diversity of sources cited in respect of primate work, the body of relevant findings in the mouse is largely the product of a single laboratory – Georg Keller (Friedrich Miescher Institute, Basel). Using behavioural paradigms to manipulate ‘natural expectation’ (as outlined above), it primarily exploits two-photon optical imaging to examine cellular and feedback axonal activity in layers 1–3 of mouse primary visual cortex (deeper layers being inaccessible due to the nature of the optical signal). The optical technique uses fluorescent signals to monitor concurrent spiking activity (bursts, as opposed to single spikes) in a large population of neurons; many remain individually identifiable over the course of multiple recording sessions, establishing a neural correlate of progressive expectation-learning behaviour. Direct comparison of findings between mouse and monkey is stymied, to a degree, by systematic differences in the laminar organisation of cortex in the two species. The supragranular layers of primate are disproportionately expanded, formed by novel developmental mechanisms and endowed with a greater diversity of glutamatergic cell types (Dehay et al., 2015; Cortay et al., 2020; Berg et al., 2021). One consequence is that the differential laminar origins of FF and FB connections between areas, that serves as a metric of hierarchical relations in the monkey, is obscure in the mouse (necessitating alternative systems based upon laminar terminations (Harris et al., 2019; D'Souza et al., 2022); see Shipp and Friston (2023) for consideration of how this bears upon hierarchical systematics of predictive coding). Irrespective of cortical lamination, predictive processing in mouse V1 does appear to employ several classes of neuron with functions to match the computational units shown in Figure 2 – although, quixotically, the first to be explicitly characterised might seem something of a misfit.

### 5.1 Prediction of optic flow speed

**Figure 9:**
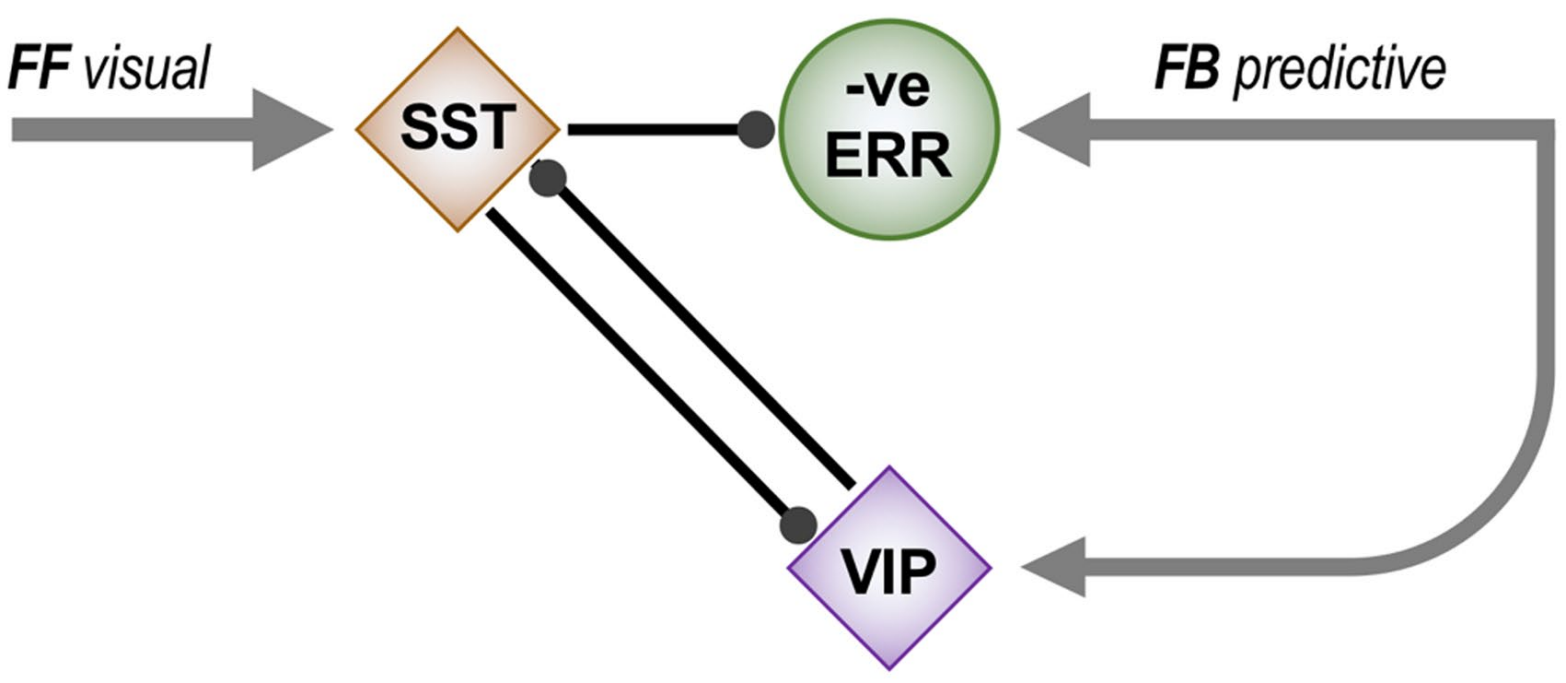
Microcircuit for generating a negative error neuron. This circuit diagram summarises findings regarding the responses of four types of interneuron to optic flow stimuli in a virtual tunnel, once the properties of (excitatory) negative error neurons had been established (Attinger et al., 2017). Two types of interneuron were primarily implicated. SST (somatostatin expressing) interneurons showed the highest correlation of activity with visual flow, whilst VIP (vasoactive intestinal polypeptide expressing) interneurons responded to ‘mismatch’, i.e., a short cessation of visual flow whilst running. Accordingly, negative error neurons are inhibited by visual signals via SST interneurons, and activated by predictive signals of visual flow generated by running, both directly and by disinhibition via the two classes of interneuron. Arrowheads indicate excitatory contacts; ballheads indicate inhibitory contacts.

The act of running is known to enhance activity in mouse visual cortex (Niell and Stryker, 2010) but, more than that, it also appears to sculpt response properties to visual motion, such that only a fraction of neurons activate purely in accord with retinal signals. Clearly, this falls within the framework of predictive processing, as the speed and direction of locomotion is a prime predictor of the optical flow field. Experimentally, mice are set to run along a linear ‘virtual tunnel’, their movement upon a trackerball, restricted to rotate in one dimension, governing visual projection onto a toroidal screen. That ‘closed-loop’ arrangement is complemented by an ‘open-loop’ condition, where visual flow from a previous trial is projected irrespective of the mouse’s movement upon the trackerball. One of the most striking findings to emerge from this set-up was the ‘omission’, or ‘mismatch’ response, a surge of activity in a sub-set of cells occasioned by a transient (1 s) pause in motion projection whilst the mouse was running in closed-loop. Further investigation, exploiting the loosening of parameters offered by running in open-loop, established that the signals from this class of neuron accurately scaled with the speed difference between predicted and visible flow, when the former was faster (Keller et al., 2012; Zmarz and Keller, 2016). A clear implication is that such neurons are, respectively, excited and inhibited by descending predictive and ascending visual signals, and a mechanism for the latter was revealed by recording from identified classes of interneurons under the same conditions. SST (somatostatin) interneurons in particular were found to be robustly activated by visual drive, with VIP (vasoactive intestinal polypeptide) interneurons also participating, according to a simple microcircuit shown in Figure 9 and Attinger et al. (2017).

Logically, an omission or mismatch response qualifies as a negative error signal [prediction – sensory signal] (Attinger et al., 2017; Keller and Mrsic-Flogel, 2018), reversing the standard formulation of error [actual quantity – prediction] prescribed by PC algorithms. From a neural perspective, this is simply pragmatic, a necessary means of producing an error signal that corrects over-prediction – given that a ‘standard’ error neuron, like all cortical neurons, has a low baseline rate, and so cannot generate negative spiking; indeed this is a recognised bug in schemes for neural implementation of PC algorithms (Kogo and Trengove, 2015). In this case the FF mismatch response should act upon frontal cortex to adapt the speed of visual flow predicted by a given level of locomotor activity – as appropriate in the real world, for instance, if the flow-speed were over-predicted due to a change in the environmental distance of the nearest surround surfaces. However, the concept of negative error and its corollary, predictive excitation, appears paradoxical with regard to the construct of predictive suppression that many studies have taken as a kind of *leitmotif* for predictive coding theory. This issue is resumed in Discussion, below.

The equation of the omission response with an error signal has been questioned in respect of an alternative interpretation, that halting motion elicits a generic visual OFF response, here enhanced by locomotion (Muzzu and Saleem, 2021). The counter-argument to that emphasises how the omission response is intimately coupled to recent visuomotor experience (Vasilevskaya et al., 2023); in particular, the omission response can be elicited by so called ‘playback halts’ (brief cessation of optic flow in the open-loop condition) and is enhanced when this occurs while the mouse is running, but never achieves the same magnitude as in the closed loop condition. Furthermore, the playback halt response progressively declines with a half-life of a few minutes throughout the course of the open-loop session, i.e., the longer the interval from the last experience of verisimilitude in the closed-loop condition. To add to this debate, these observations are readily interpretable in the context of visual learning as a form of precision regulation. The open-loop condition renders the sensory feedback from locomotion unreliable; adjustments to the visuomotor model generating predictions, and to locomotor speed, both fail to result in predictable consequences. Hence precision – the regulatory gain applied to the error signal – declines in line with gPC theory, as outlined previously.

**Figure 10:**
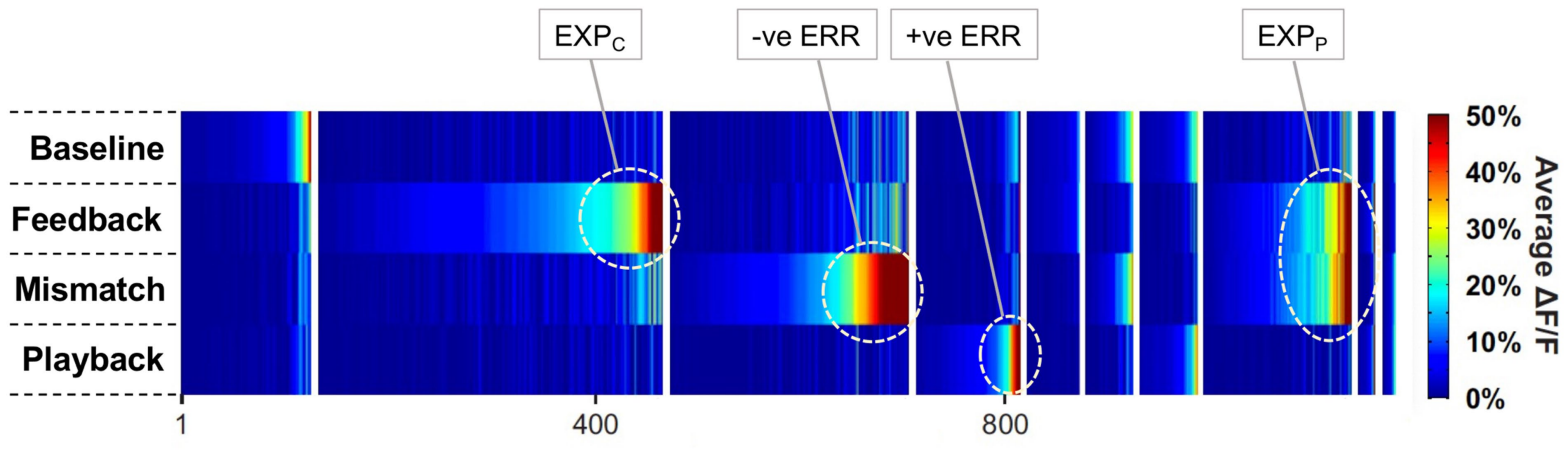
Putative functional identities of mouse V1 neurons activated during running and optic flow. The graphic shows the peak response, averaged across trials, of 1,171 neurons in layer 2/3 of mouse V1, in four conditions with varied combinations of running/static locomotion, and presence/absence of simulated optic flow (Keller et al., 2012). Neurons in the first four groups (from left, denoted by vertical blank dividers) responded primarily in one condition; those in the next six groups responded equally in two conditions; unresponsive neurons are not shown. Baseline: static with absent optic flow; Feedback: running with optic flow; Mismatch: brief cessation of optic flow whilst running; Playback: static with optic flow. Labels in the top line are provisional assignments of computational role according to predictive coding theory. +ve ERR: standard error neurons activated by visual flow, and undergoing predictive suppression by running; −ve ERR: negative error neurons undergoing predictive excitation by running, and suppressed by optic flow. EXP_C_: coding expectation neurons activated by optic flow, and concurrently disinhibited by running-induced predictive suppression of standard error neurons. EXP_P_: predictive expectation neurons carrying flow-predicting signals from frontal cortex, hence active during running irrespective of the presence or absence of optic flow. Reproduced by permission of Elsevier.

**Figure 11:**
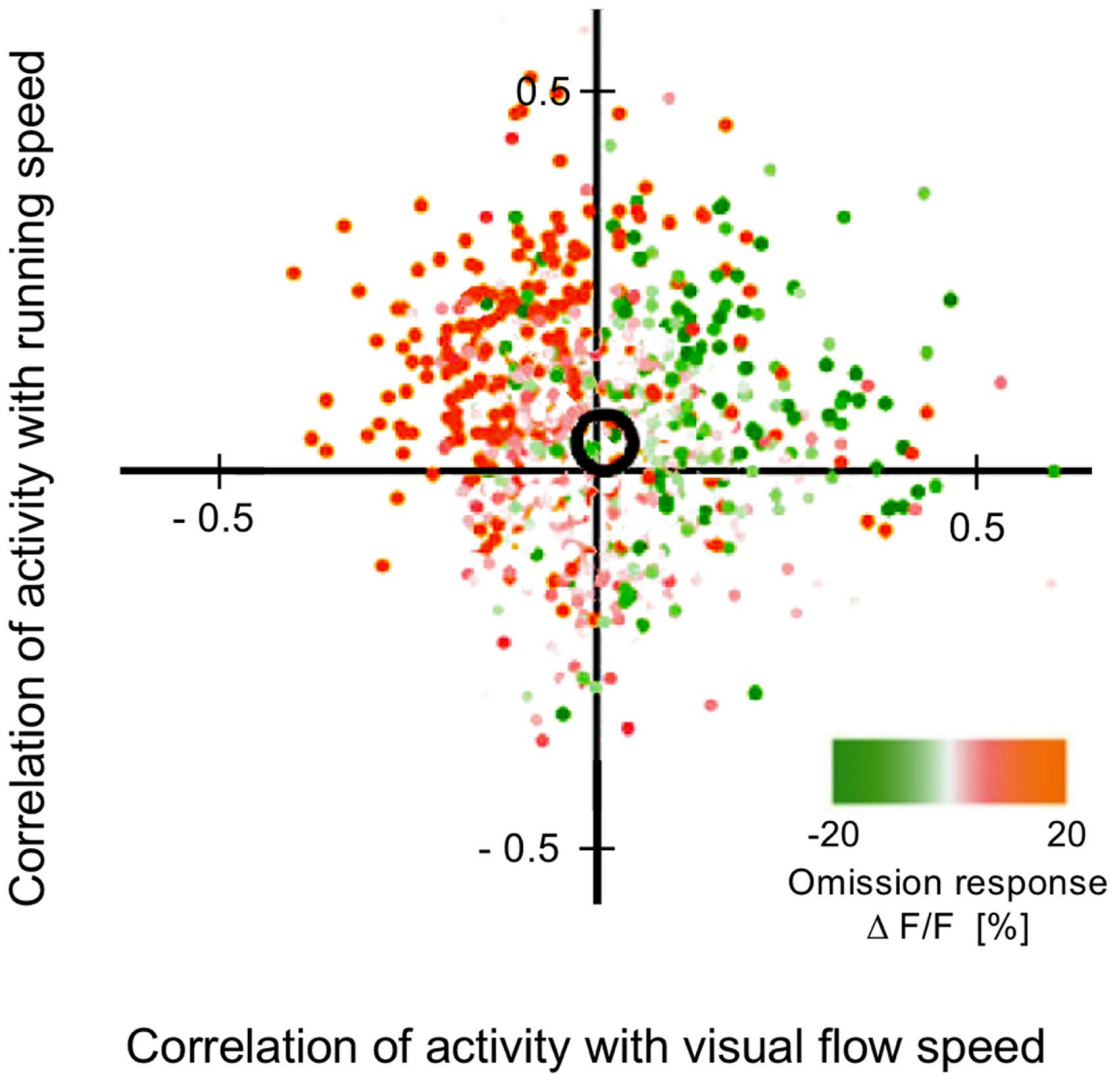
Co-determination of activity of mouse V1 neurons by running speed and optic flow speed. The graphic shows the joint correlation of activity of 2,259 neurons in layer 2/3 of V1 with running speed and optic flow speed, as determined during open loop conditions of mice running in a virtual tunnel (Attinger et al., 2017). In regard to running (vertical axis), predictive activation is more common than predictive suppression, whereas the balance between excitation and inhibition by visual flow is more even; the black circle marks the bi-dimensional mean correlation. Colour coding indicates a third classification, the nature of response to stimulus omission (a brief cessation of optic flow whilst running) roughly aligning with flow speed correlation. The authors identified cells in the top-left quadrant as negative error neurons (predictive activation from running, suppression by optic flow). Logically, therefore, cells in the lower-right quadrant exhibiting the opposite properties (predictive suppression by running, excitation by optic flow) should be positive error neurons. Cells in the top-right quadrant could qualify as EXP_C_ neurons, on account of activation by both flow and running; direct excitation by optic flow, and running induced disinhibition via positive error cells. By symmetry, cells in the lower left quadrant logically qualify as ‘negative’ EXP_C_ neurons; to rationalise this notion, they might hypothetically be neurons excited by a reverse, receding optic flow field, and by backward locomotion – and jointly suppressed by forward locomotion and an advancing flow field, as tested. Neurons conveying a flow-predictive signal from frontal areas might also populate the diagram, congregating about the vertical axis with near zero optic flow corelation. Reproduced by permission of Elsevier.

The co-existence of standard, positive error neurons for flow speed prediction was acknowledged in theory (Attinger et al., 2017; Keller and Mrsic-Flogel, 2018) before being empirically confirmed by a study resorting to intracellular recording from behaving mouse V1 in the same virtual tunnel set-up (Jordan and Keller, 2020). Monitoring subthreshold changes in membrane potential, it found one set of superficial layer neurons that tended to depolarise with increasing visual flow speed, and hyperpolarise with increasing locomotor speed (implied positive error neurons), and another that did exactly the reverse (implied negative error neurons). Moreover, recent work suggests that these two types are further distinguished by belonging to two different transcriptomic classes of excitatory neuron, of which three altogether have been identified in layers 2/3 of mouse V1 (Tasic et al., 2018; O’Toole et al., 2023). In retrospect, positive error neurons were cryptically present in earlier datasets, forming a covert minority fraction, whereas other classes – varieties of EXP neuron – were possibly hiding in plain sight, unrecognised by the heuristics of predictive processing applied by these authors. Figures 10, 11 discuss two examples. In contrast to the superficial neurons, deeper layers of V1 (principally layer 5, sampled by the electrodes used for intracellular recording) integrated visual flow and locomotor speed in non-opponent fashion, both commonly exerting a depolarising effect at greater speeds (Jordan and Keller, 2020). This was closer to earlier observations made elsewhere, by means of a similar virtual tunnel recording strategy combined with depth electrodes (Saleem et al., 2013); it is also rather more similar to the nature of frontal predictive signals themselves, as observed in their axon terminals within V1.

Frontal terminations in mouse V1 from ACC (anterior cingulate cortex) are known to modulate visual processing in V1 and mediate attentional effects via their interaction with inhibitory circuits (Zhang S. et al., 2014). Anatomical tracing shows that this frontal projection retains topographic order and contacts diverse types of interneuron, as well as excitatory cells lying mainly in layers 2/3 and 6 (Leinweber et al., 2017). Subsequent viral transfection of the densest source of the ACC back projection with the Ca indicator GCaMP6f allowed optical monitoring of ACC axonal activity in V1, and demonstrated that the predictive signal is not purely a form of motor efference-copy (Leinweber et al., 2017). Some 30% of axons initiated activity well ahead (upto 1 s) of running onset in the closed loop condition; the remainder progressively came onstream upto 1 s after running onset. This activity increased with running speed, but was reduced in full darkness. In open-loop, visual flow deferred from running onset was seen to augment activity with a latency of 1 s. By contrast, the presence of visual flow prior to running onset had the opposite effect, reducing activity. Presumably therefore, the visuomotor model operated by ACC enjoys a re-entrant loop with visual cortex, such that current visual signals integrate with motor signals in ACC to abet the prediction of future visual signals.

### 5.2 Prediction of spatially localised features

Another means of exploiting the virtual tunnel set-up is to examine predictions based upon distance travelled, in this case predictions of the orientation of gratings encountered at fixed virtual positions en route to a recognisable, rewarded end point (Fiser et al., 2016). Orthogonal gratings ‘A’ and ‘B’ were presented in a regular A-B-A-B-X sequence (where X, initially set to A, subsequently underwent conditional variability to test responses to unpredicted stimuli). An initial observation was that a subset of visual, orientation-tuned neurons that were selective for either A or B responded unequally at different locations (e.g., A1 vs. A2), such that a neural decoding algorithm was able to predict tunnel location from population activity. As the mice gained experience of traversing the tunnel in multiple trials across multiple sessions, these visual neurons progressively enhanced their response magnitude. Furthermore a second class of neurons emerged – ‘prediction neurons’ – likewise selective for either A or B, but whose activity initiated shortly before the grating was physically displayed; with further traversals, these neurons’ responses also gained in magnitude. These two classes of neuron might thus be characterised as orientation-specific ‘predictors’ and ‘detectors’, as further underlined by testing across repeated sessions in which the fifth sequential grating, ‘X’, was 90% A and 10% B across trials (or the reverse, 10% A and 90% B, in a later set of sessions). Once accustomed to this conditionality, a detector neuron simply continued to respond appropriately to the grating presented. By contrast, a predictor neuron’s response anticipated the expected grating, irrespective of the grating actually presented; for example the mean activity of B predictors encountering X in a 90% B condition was equal for X = A and X = B, but marginally less than the activity at B2 and B4 at earlier locations in the tunnel, where B was 100% expected.

**Figure 12:**
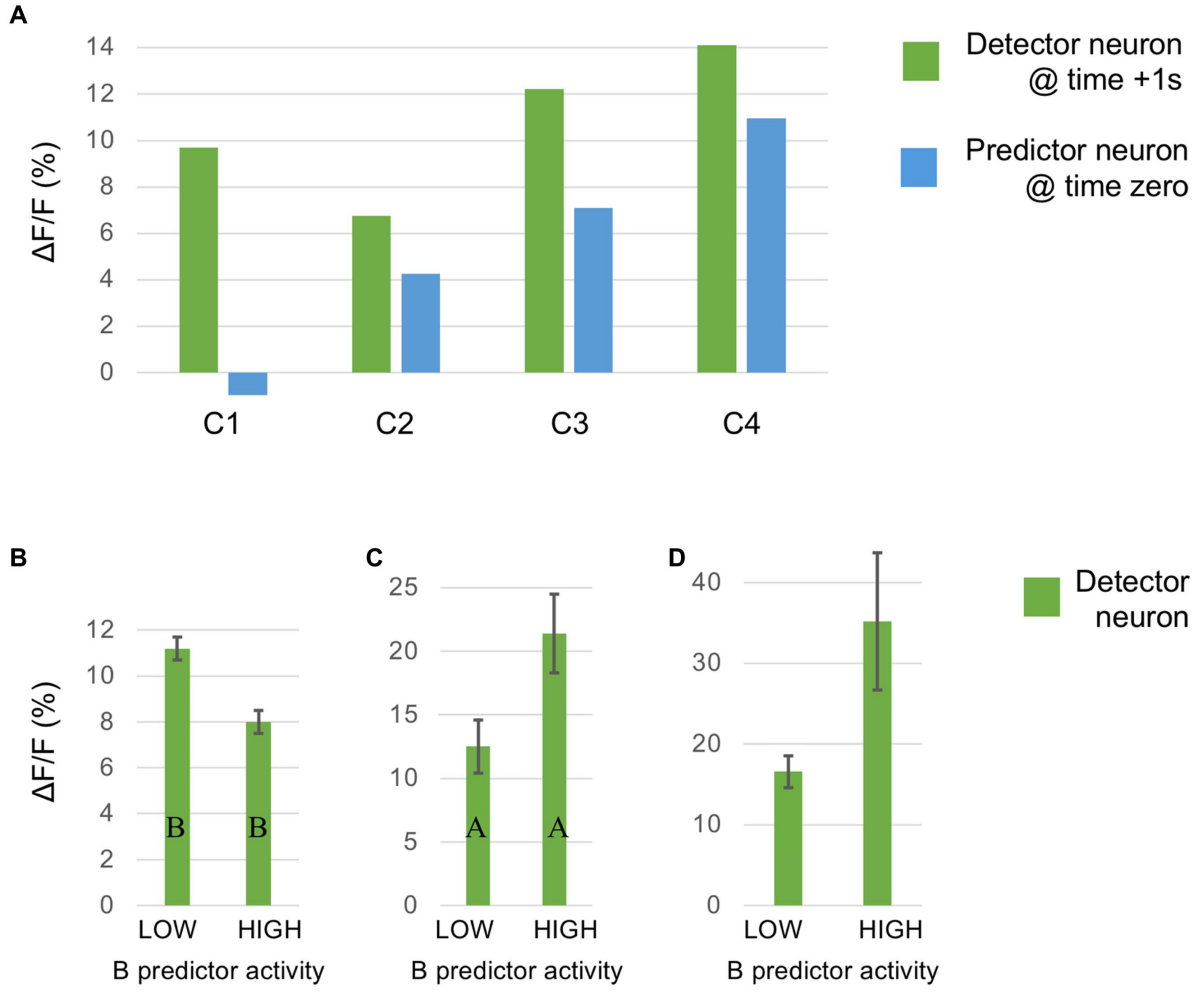
Evidence of interactions between visual ‘predictor’ and ‘detector’ neurons in mouse V1, from the study of Fiser et al. (2016). Mice traversing a virtual tunnel encountered a sequence of five gratings: A-B-A-B-X, where X varied across conditions C1-C5: X = 100% A (C1); 90% B, 10% A (C2); 100% B (C3); 10% A, 90% B (C4); 10% null, 90% B (C5). Individual neuron responses were traced across this fixed order of conditions, and exhibited visual learning as mice progressively gained tunnel experience. **(A)** The relative, mean responses of the two classes of neuron across C1-C4 (pooling A and B selectivities). Predictor neurons sampled at stimulus onset, detector neurons sampled 1 s post-onset. **(B–D)** Trial-by-trial, detector neuron responses are contingent upon prior activity of predictor neurons. **(B)** Mean responses of B-selective detector cells to B-grating stimulus ‘B5’, in condition 4, in two subsets of trials: those with high and low predictive activity, respectively the top and bottom 20% of trials according to the mean, prior activity of B-predictor neurons. B-detector neuron activity is sampled 0.5–1.0 s post stimulus onset. **(C)** Mean responses of A-selective detector neurons to unexpected A-grating stimulus A5 in condition 4, in subsets of trials with high and low predictive activity (respectively the top and bottom 50% with respect to prior B-predictor neuron activity). A-detector neuron activity is sampled 0.5–1.0 s post stimulus onset. **(D)** Mean ‘omission’ responses to the unexpected absence of B-grating stimulus B5 in condition 5, in subsets of trials with high and low predictive activity (respectively the top and bottom 50% with respect to prior B-predictor neuron activity). Omission activity is sampled 0.5–1.0 s post stimulus omission (i.e., post-onset, as timed in non-omission trials).

In terms of the computational unit classes envisaged in Figure 2, predictor neurons may equate to EXP_P_ units, and detector neurons might comprise a mixture of EXP_C_ and ERR units. There should therefore be interactions between them, and several forms were indeed observed. Firstly, as noted above, the average magnitude of activation of both classes grew larger across successive conditions. But, interrupting this trend, the emergence of predictor neurons in condition 2 was marked by a decrement of detector neurons’ activity, with incremental activity resuming through conditions 3 and 4 (Figure 12A). This implies that predictor neurons had a suppressive effect upon the net activity of detector neurons. A second indication of such suppression was that, across trials in the same condition, higher activity in (say, B) predictor neurons was associated with lower activity amongst B detector neurons, and vice versa (Figure 12B). The inference, again, is that a substantial proportion of detector neurons generate a standard, positive error signal.

There are two further findings in regard to interactions between predictor and detector neuron population activity that are relevant to a lingering issue raised above, the status of a negative error signal within predictive coding theory. By way of introduction, it is helpful to refer to interactions between V1 and LGN (as documented in the cat; see Shipp (2016) for a fuller account). LGN relay neurons are held to be ERR units. Their receptive fields are either ON or OFF selective, and they receive ON and OFF backward input from V1, interpreted as predictions of local contrast. The ON predictions suppress ON relay cell activity, and likewise OFF suppresses OFF, consistent with a positive ERR unit function; however, acting with equal magnitude, ON predictions also enhance OFF relay cell activity, and OFF predictions enhance ON relay cell activity (Wang et al., 2006). In other words, a prediction may both be subtracted from its home channel, and added to an opposite channel, such that LGN relay cells combine the roles of positive and negative error signalling. Fiser et al. (2016) report a comparable observation, in regard to the response of detector neurons to unexpected stimuli in the condition described above, where fifth grating X was 90% B, and 10% A across trials. They found that the mean response of A detectors to unexpected A5 was higher in trials where the shortly preceding mean activity of B predictors was also high, and lower when it was lower (Figure 12C). Empirically, this implies that B-predictive signals act to excite A detector units, and theoretically it is consistent with B-predictive signals enhancing the activity of our previously inferred subset of A-detectors that are positive ERR units, potentially endowing them with negative error signalling capacity.

The second finding of interest concerns an omission response, occasioned by a condition in which the fifth grating was 90% B, and 10% absent. Stimulus omission created an unusually strong response, with a small minority (2.3%) of neurons responding exclusively in these trials. Selective analysis of these omission-sensitive neurons showed an inverse relationship to concurrent B predictive activity: more omission activity on trials in which B predictive activity was high, and less when it was low (Figure 12D). Because omission neurons were active only in respect of an absent stimulus, they were not classified as A or B selective. However, following the logic above, their pattern of activity was possibly consistent with a variant of ‘A-detector’ error neuron subject to atypically strong excitation from B-predictors, but with nugatory visual excitatory drive. Its role would be to nullify any expectation-induced representation of B in higher centres.

**Figure 13:**
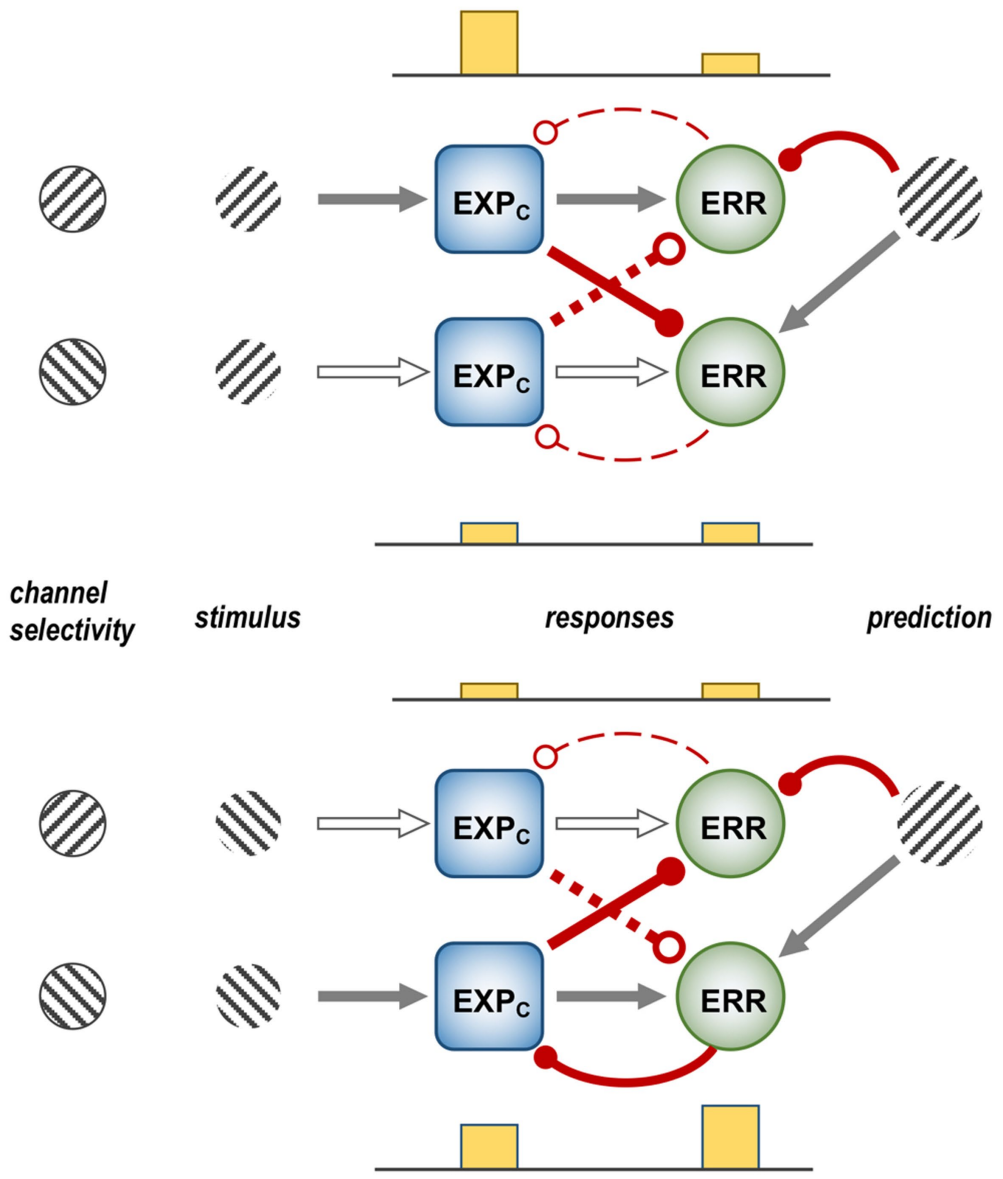
The ‘crossover’ model of error synthesis. The crossover model has two founding principles: (i) that negative error units exist, and are necessary for correcting overprediction (of prothetic variables) or misprediction of qualitative variables (such as orientation); (ii) that, for reasons of economy, a single error unit might combine both positive and negative error signalling. Each schematic shows a pair of channels selective for orthogonal orientations (where a ‘channel’ comprises an EXP_C_ unit and an ERR unit with similar tuning), their elementary circuitry, and their stimulus responses. The prediction is shown to suppress the ERR unit in its ‘home’ channel, and excite the ERR unit in the opponent channel. Suppressive cross-connections (EXP_C_ to ERR) have been added between the channels to replicate the finding in mouse V1 that negative error neurons are inhibited by visual stimuli. Upper schematic: the prediction is consonant with the stimulus, suppresses the ERR unit in its home channel, and acts to excite the ERR unit in the opponent channel. The home EXP unit, activated by the visual stimulus, is also disinhibited following predictive suppression of the home ERR unit; it suppresses activity of the opponent ERR unit. Hence neither ERR unit is active, and the prediction (i.e., representation) of orientation present at a higher level is maintained. Lower schematic: prediction of the same orientation, now a misprediction of the stimulus, continues to act in the same way upon the two ERR units; but the home EXP_C_ unit is inactive, and the opponent EXP_C_ unit co-excites the opponent ERR unit. In consequence (but not shown) forward transmission of the opponent error signal switches the orientation represented at a higher level, that in turn leads to predictive suppression of the error signal in that channel, and elevated activity of the EXP_C_ unit through disinhibition. Arrows versus ballheads indicate excitatory versus inhibitory connections; Solid versus open or dashed connections indicate active versus inactive connections.

In developing this model of ‘crossover’ synthesis of error signals (see Figure 13) it is noteworthy that A and B were orthogonal orientations, and it is a reasonable caveat that the model is more appropriate for basic visual attributes encoded as opposites (e.g., contrast, orientation, direction, opponent colours) and less so for complex object properties analysed by inferotemporal cortex (see Discussion). If so, might the same crossover systematics exist in the mechanisms predicting optic flow, and the observed omission responses on the part of negative error neurons, discussed above? The relevant experimental observations are lacking, reflecting an obvious behavioural asymmetry: a receding flow field, occasioned as an animal scuttles backward, is rarely experienced. Hypothetically the distinctive couplings shown by positive and negative error neurons for hyperpolarisation/depolarisation effected by faster/slower optic flow speed and locomotor speed (Jordan and Keller, 2020) might all be reversed in the eventuality of a receding flow field. Whether this is actually the case remains to be tested.

Finally, Fiser et al. (2016) were also able to examine the activity of ACC axon terminals in V1, and again distinguished predictor and visual (or detector, as termed here) variants. As with cellular activity, predictive signals were absent on initial tunnel traversals and developed with accumulated experience; detector signals also gained in magnitude, and also developed a modest initial phase of pre-stimulus predictive activity. Probing these responses with unexpected stimuli again proved instructive. Predictor axon activity reflected the conditional probabilities of 90% B, 10% A for the fifth grating, in that B-predictors were marginally less active in comparison to B2 and B4, and A-predictors gave a tiny response, much less than to A1 and A3, but still significant. B-detector axons responded comparably to B5 as to B2 and B4; the initial (predictive) phase of their response to unexpected A at location 5 was also comparable, but declined rapidly following stimulus onset of A5. In the same condition, A-detector axons gave no response to B5, whilst their response to A5 lacked the initial predictive phase, and was smaller than the response to A1 and A3. One way of rationalising this duality of behaviour is to infer that predictor signals represent a temporal-forecast component of prediction, and detector signals represent the current hypothesis of what is being seen, albeit with a modest, predictive ‘jump-start’.

## 6 Discussion

This review examines the neural implementation of predictive coding at the level of basic circuit components, the properties of excitatory pyramid neurons in particular assessed from a physiological perspective. It is necessary to work from a simple circuit model, informed by anatomical data – the purview here being the primate visual system (Figure 2). A parallel literature is available in the auditory domain (Chao et al., 2018; Heilbron and Chait, 2018; Walsh et al., 2020); across species, work in the mouse visual system leads the way in many technical respects impossible or impracticable to replicate in primates and demanding parallel consideration, whatever the uncertainties with regard to primate homology. The Introduction refers to a ‘voluminous literature’: sifting through it, as best one can, there is little or nothing to violate the basic precepts of predictive coding (after sufficient scrutiny). And there is much that can be rationalised in the light of predictive processing, often a more coherent and parsimonious rationale than ingenious alternative interpretations. From this perspective, predictive coding theory can rightly claim solid foundations in basic neuroscience. Although how it might work, exactly, remains richly perplexing.

Focusing initially upon primate studies that directly address predictive coding theory, there is evidence linking FF error signalling to gamma oscillatory activity in the superficial layers (Bastos et al., 2020; Esmailpour et al., 2022), and varied findings regarding timing: where stimulus expectation precedes stimulus onset, error signalling is reportedly virtually immediate, from peak response onward (Meyer and Olson, 2011; Schwiedrzik and Freiwald, 2017); where expectation is the outcome of viewing the stimulus, it is more delayed (Issa et al., 2018). Notably however, multi-factor regression modelling of population responses (as opposed to subtraction of population response curves) suggests that error signalling can be delayed even when expectation is pre-formed (Figure 6; Bell et al., 2016) – possibly a more secure conclusion, given the explicit efforts to exclude any confounding effects of repetition suppression in this study. Prediction signals, by contrast, are linked to beta activity in the deep layers. In apex frontal areas, this should correspond to the activity of FB, EXP_P_ neurons; in intermediate areas, for instance V4 (Bastos et al., 2020) or IT (Esmailpour et al., 2022) the signals might associate with both FB and intrinsic classes; these co-populate the deep layers according to the template model (Figure 2) and cannot be distinguished by measures of gross population activity such as LFP recordings. Looking to complementary findings in human cortex, a high-resolution fMRI study that cued expectation of one or other orthogonal gratings succeeded in detecting predictive signals in V1, specifically in the deep layers (Aitken et al., 2020). This might preferentially implicate activity in intrinsic EXP_P_ neurons, as extrinsic EXP_P_ neurons in V1 communicate backward to LGN, that lacks orientation selectivity.

As outlined above, experimental paradigms exploit multiple (natural versus statistical) means to manipulate expectation, that may well differ in their origins amongst the brain’s cortical and subcortical systems, and in their subsequent modes of transmission and mechanisms of action. Adopting a broad outlook, it is of interest to draw a loose parallel between monkey and mouse observations pertaining to a binary origin of predictions in frontal cortex. Frontal cortex in the monkey was inferred to issue predictive signals solely from the deep layers prior to an expected stimulus onset, and more so from the superficial layers once the stimulus was visible (Bastos et al., 2020). Frontal ACC axons in mouse, observed in V1 (and hence of unknown laminar origin), were classified as either predictive or visual (Fiser et al., 2016). The former were activated by expectation, ahead of and irrespective of whatever stimulus was presented; the latter initiated activity just moments ahead of stimulus presentation, that either grew more robust or died away contingent upon stimulus identity. Thus, in both cases, the distinction looks to be between (pre-stimulus) expectation as temporal forecast, and (post-stimulus) expectation as current hypothesis – indeed, it would be surprising if a single neural mechanism were to exhibit such dual functionality.

Coding EXP neurons (EXP_C_ – so named for activity hypothetically forming the direct substrate of perceptual experience) have proved more reclusive, identified by default as non-ERR neurons; either explicitly, e.g., in IT cortex of primate (Issa et al., 2018), or by retrospective analysis as essayed here for the mouse (Figures 10, 11). Potentially, the clearest demarcation of EXP from ERR neurons has been achieved by means of the binocular rivalry paradigm (Logothetis and Schall, 1989; Leopold and Logothetis, 1996). This (or other bi-stable percepts, not contingent on ocular rivalry), used in conjunction with multilaminar electrodes could prove a potent vehicle for testing the elemental neural interactions comprising the gPC template model, particularly with regard to their relative timing. The inverse pattern of activity of inferred EXP and ERR neurons observed in V4 is consistent with the nature of their proposed reciprocal connections (Figure 8). However, the change in activity triggered by the higher level switch in visual cognition should be registered first by deep EXP_P_ neurons, and then communicated to superficial EXP_C_ neurons via superficial ERR neurons, phases that might all be captured by concurrent multilaminar recordings.

Many open questions remain regarding the principles whereby predictions are directed to and distributed among populations of ERR neurons. The revelation of negative error neurons, excited by predictions and signalling the degree to which a prediction may be an overestimate (Keller and Mrsic-Flogel, 2018), challenges the construct of ‘predictive suppression’ which informs many experimental approaches. If predictive excitation is equally common, ‘predictive error-discovery’ would be a more fitting appellation. However there is scant empirical verification of predictive excitation outside of optic flow processing in mouse V1. Speed is a prothetic variable – might its quantitative metric be processed differently to qualitative variables such as orientation, direction or colour? The only other frank evidence for negative error signals comes from LGN neurons processing ON and OFF contrast – again, a prothetic variable. Given that the LGN has a far simpler computational architecture, it may not be a good model of the cortex, but the point of interest about LGN neurons is that, interpreted as error neurons, they each combine positive and negative error signalling (Wang et al., 2006; Jehee and Ballard, 2009; Shipp, 2016). This can be generalised to a ‘cross-over’ model of error synthesis (Figure 13), whereby a prediction does not merely suppress error signalling within its own ‘home’ channel, but also enhances error-signalling within the opponent channel. As documented above, mice V1 neurons tuned to orthogonal gratings showed some predictive response properties that were consistent with this idea (Fiser et al., 2016). There is also an analogous fMRI finding from human subjects observing bistable rotary motion (with naturally reversing percepts of direction akin to ocular rivalry): a subset of voxels in area V5 with net tuning to one direction (when viewing non-ambiguous motion) were found to show enhanced activity in periods when subjects reported the opposite direction percept, that the authors modelled as prediction error (Weilnhammer et al., 2021).

The idea of predictive excitement also bears, in principle, upon the issue of ‘surprise enhancement’: this is the observation in IT cortex that, compared to a neutral (absent) state of expectation, one and the same stimulus can not only elicit a smaller response when expected (i.e., predictive suppression) but also a larger response when it is unexpected (Kaposvari et al., 2018; Feuerriegel et al., 2021). However, it is problematic to apply the idea of crossover error-signalling to experiments conducted in IT cortex where [with the exception of Schwiedrzik and Freiwald (2017)] there is no overt conception of how dimensions of neural encoding relate to image variables in the stimulus ensemble. The problem being, in other words, that ‘home’ and ‘opponent’ neural channels for any given stimulus are not readily recognisable. This matter was approached empirically by the pioneering study of error signalling in IT cortex where, for each neuron studied, stimuli were ranked from grade 1 to 6 by the magnitude of the response elicited (Meyer and Olson, 2011; Ramachandran et al., 2017). In this statistical learning paradigm, each leading stimulus predicted a specific trailing stimulus, so it was possible to assess whether expectation of grade 1 versus grade 6 had any effect upon a neuron’s response to an unexpected stimulus (one of grade 2–5). The fact that it did not led the authors to conclude that the nature of the error signals they described was not entirely compliant with predictive coding theory.

The undercurrent of thinking here is that any given prediction should exert a suppressive effect upon an error neuron in proportion to the response magnitude of that error neuron to the particular predicted stimulus – and that this suppression should be apparent (as the appropriate proportional reduction) in the response to any stimulus, predicted or not. In fact, the IT dataset was more consistent with the generalisation that predictive suppression only occurred when the stimulus presented was *the* predicted stimulus, and not any other (note that all stimuli employed were highly distinctive). Taken at face value, this finding is not altogether compliant with a crossover model of error synthesis. Even so, it might better be taken as a refinement rather than violation of predictive coding heuristics – and, perhaps, a challenge to devise a ‘gating’ neural mechanism for matching descending predictive signals against ascending sensory signals, before allowing predictive signals access to error neurons. The more realistic conclusion is, of course, that there is simply a dearth of relevant experimental data, and that attempting to infer general principles of neural mechanisms implementing the gPC algorithm across different cortical areas and species studied by extraordinarily different means of manipulating expectation remains very much a work-in-progress.

## Author contributions

SS: Conceptualization, Visualization, Writing – original draft, Writing – review & editing.

## References

[ref1] Adams W. J. Graf E. W. Ernst M. O. (2004). Experience can change the 'light-from-above' prior. Nat. Neurosci. 7, 1057–1058. doi: 10.1038/nn1312, PMID: 15361877

[ref2] Adams R. A. Shipp S. Friston K. J. (2013). Predictions not commands: active inference in the motor system. Brain Struct. Funct. 218, 611–643. doi: 10.1007/s00429-012-0475-5, PMID: 23129312 PMC3637647

[ref3] Aitken F. Menelaou G. Warrington O. Koolschijn R. S. Corbin N. Callaghan M. F.. (2020). Prior expectations evoke stimulus-specific activity in the deep layers of the primary visual cortex. PLoS Biol. 18:e3001023. doi: 10.1371/journal.pbio.3001023, PMID: 33284791 PMC7746273

[ref4] Akam T. Kullmann D. M. (2014). Oscillatory multiplexing of population codes for selective communication in the mammalian brain. Nat. Rev. Neurosci. 15, 111–122. doi: 10.1038/nrn3668, PMID: 24434912 PMC4724886

[ref5] Alink A. Schwiedrzik C. M. Kohler A. Singer W. Muckli L. (2010). Stimulus predictability reduces responses in primary visual cortex. J. Neurosci. 30, 2960–2966. doi: 10.1523/JNEUROSCI.3730-10.2010, PMID: 20181593 PMC6633950

[ref6] Alonso J. M. Martinez L. M. (1998). Functional connectivity between simple cells and complex cells in cat striate cortex. Nat. Neurosci. 1, 395–403. doi: 10.1038/1609, PMID: 10196530

[ref7] Attinger A. Wang B. Keller G. B. (2017). Visuomotor coupling shapes the functional development of mouse visual cortex. Cells 169, 1291–1302.e1214. doi: 10.1016/j.cell.2017.05.023 28602353

[ref8] Auksztulewicz R. Friston K. (2016). Repetition suppression and its contextual determinants in predictive coding. Cortex 80, 125–140. doi: 10.1016/j.cortex.2015.11.024, PMID: 26861557 PMC5405056

[ref9] Bair W. Cavanaugh J. R. Movshon J. A. (2003). Time course and time-distance relationships for surround suppression in macaque V1 neurons. J. Neurosci. 23, 7690–7701. doi: 10.1523/JNEUROSCI.23-20-07690.2003, PMID: 12930809 PMC6740744

[ref10] Barberini C. L. Cohen M. R. Wandell B. A. Newsome W. T. (2005). Cone signal interactions in direction-selective neurons in the middle temporal visual area. J. Vis. 5, 603–621. doi: 10.1167/5.7.1, PMID: 16231996

[ref11] Bastos A. M. Litvak V. Moran R. Bosman C. A. Fries P. Friston K. J. (2015a). A DCM study of spectral asymmetries in feedforward and feedback connections between visual areas V1 and V4 in the monkey. NeuroImage 108, 460–475. doi: 10.1016/j.neuroimage.2014.12.081, PMID: 25585017 PMC4334664

[ref12] Bastos A. M. Lundqvist M. Waite A. S. Kopell N. Miller E. K. (2020). Layer and rhythm specificity for predictive routing. Proc. Natl. Acad. Sci. U. S. A. 117, 31459–31469. doi: 10.1073/pnas.2014868117, PMID: 33229572 PMC7733827

[ref13] Bastos A. M. Usrey W. M. Adams R. A. Mangun G. R. Fries P. Friston K. J. (2012). Canonical microcircuits for predictive coding. Neuron 76, 695–711. doi: 10.1016/j.neuron.2012.10.038, PMID: 23177956 PMC3777738

[ref14] Bastos A. M. Vezoli J. Bosman C. A. Schoffelen J. M. Oostenveld R. Dowdall J. R.. (2015b). Visual areas exert feedforward and feedback influences through distinct frequency channels. Neuron 85, 390–401. doi: 10.1016/j.neuron.2014.12.018, PMID: 25556836

[ref15] Bauer M. Stenner M. P. Friston K. J. Dolan R. J. (2014). Attentional modulation of alpha/beta and gamma oscillations reflect functionally distinct processes. J. Neurosci. 34, 16117–16125. doi: 10.1523/jneurosci.3474-13.2014, PMID: 25429152 PMC4244475

[ref16] Bell A. H. Summerfield C. Morin E. L. Malecek N. J. Ungerleider L. G. (2016). Encoding of stimulus probability in macaque inferior temporal cortex. Curr. Biol. 26, 2280–2290. doi: 10.1016/j.cub.2016.07.007, PMID: 27524483 PMC5021632

[ref17] Bell A. H. Summerfield C. Morin E. L. Malecek N. J. Ungerleider L. G. (2017). Reply to Vinken and Vogels. Curr. Biol. 27, R1212–R1213. doi: 10.1016/j.cub.2017.09.021, PMID: 29161557 PMC5747320

[ref18] Berezovskii V. K. Nassi J. J. Born R. T. (2011). Segregation of feedforward and feedback projections in mouse visual cortex. J. Comp. Neurol. 519, 3672–3683. doi: 10.1002/cne.22675, PMID: 21618232 PMC3219532

[ref19] Berg J. Sorensen S. A. Ting J. T. Miller J. A. Chartrand T. Buchin A.. (2021). Human neocortical expansion involves glutamatergic neuron diversification. Nature 598, 151–158. doi: 10.1038/s41586-021-03813-8, PMID: 34616067 PMC8494638

[ref20] Bijanzadeh M. Nurminen L. Merlin S. Clark A. M. Angelucci A. (2018). Distinct laminar processing of local and global context in primate primary visual cortex. Neuron 100, 259–274.e4. doi: 10.1016/j.neuron.2018.08.020, PMID: 30220509 PMC6279245

[ref21] Blasdel G. G. Fitzpatrick D. (1984). Physiological organization of layer 4 in macaque striate cortex. J. Neurosci. 4, 880–895. doi: 10.1523/jneurosci.04-03-00880.1984, PMID: 6200586 PMC6564839

[ref22] Blom T. Feuerriegel D. Johnson P. Bode S. Hogendoorn H. (2020). Predictions drive neural representations of visual events ahead of incoming sensory information. Proc. Natl. Acad. Sci. U. S. A. 117, 7510–7515. doi: 10.1073/pnas.1917777117, PMID: 32179666 PMC7132318

[ref23] Bonnefond M. Kastner S. Jensen O. (2017). Communication between brain areas based on nested oscillations. eNeuro 4, ENEURO.0153–ENEU16.2017. doi: 10.1523/eneuro.0153-16.2017, PMID: 28374013 PMC5367085

[ref24] Borra E. Rockland K. S. (2011). Projections to early visual areas V1 and V2 in the calcarine fissure from parietal association areas in the macaque. Front. Neuroanat. 5:35. doi: 10.3389/fnana.2011.00035, PMID: 21734867 PMC3123769

[ref25] Bosking W. H. Zhang Y. Schofield B. Fitzpatrick D. (1997). Orientation selectivity and the arrangement of horizontal connections in tree shrew striate cortex. J. Neurosci. 17, 2112–2127. doi: 10.1523/JNEUROSCI.17-06-02112.1997 9045738 PMC6793759

[ref26] Bosman C. A. Schoffelen J. M. Brunet N. Oostenveld R. Bastos A. M. Womelsdorf T.. (2012). Attentional stimulus selection through selective synchronization between monkey visual areas. Neuron 75, 875–888. doi: 10.1016/j.neuron.2012.06.037, PMID: 22958827 PMC3457649

[ref27] Bourgeois A. Guedj C. Carrera E. Vuilleumier P. (2020). Pulvino-cortical interaction: an integrative role in the control of attention. Neurosci. Biobehav. Rev. 111, 104–113. doi: 10.1016/j.neubiorev.2020.01.005, PMID: 31972202

[ref28] Briggs F. Callaway E. M. (2001). Layer-specific input to distinct cell types in layer 6 of monkey primary visual cortex. J. Neurosci. 21, 3600–3608. doi: 10.1523/JNEUROSCI.21-10-03600.2001, PMID: 11331389 PMC1820845

[ref29] Brodski A. Paasch G. F. Helbling S. Wibral M. (2015). The faces of predictive coding. J. Neurosci. 35, 8997–9006. doi: 10.1523/jneurosci.1529-14.2015, PMID: 26085625 PMC6605164

[ref30] Buffalo E. A. Fries P. Landman R. Buschman T. J. Desimone R. (2011). Laminar differences in gamma and alpha coherence in the ventral stream. Proceedings of the National Academy of Sciences USA 108, 11262–11267. doi: 10.1073/pnas.1011284108, PMID: 21690410 PMC3131344

[ref31] Buzsaki G. Schomburg E. W. (2015). What does gamma coherence tell us about inter-regional neural communication? Nat. Neurosci. 18, 484–489. doi: 10.1038/nn.3952, PMID: 25706474 PMC4803441

[ref32] Buzsaki G. Wang X. J. (2012). Mechanisms of gamma oscillations. Annu. Rev. Neurosci. 35, 203–225. doi: 10.1146/annurev-neuro-062111-150444, PMID: 22443509 PMC4049541

[ref33] Callaway E. M. Wiser A. K. (1996). Contributions of individual layer 2-5 spiny neurons to local circuits in macaque primary visual cortex. Vis. Neurosci. 13, 907–922. doi: 10.1017/S0952523800009159, PMID: 8903033

[ref34] Chao Z. C. Takaura K. Wang L. Fujii N. Dehaene S. (2018). Large-scale cortical networks for hierarchical prediction and prediction error in the primate brain. Neuron 100, 1252–1266.e1253. doi: 10.1016/j.neuron.2018.10.004 30482692

[ref35] Chaudhuri R. Knoblauch K. Gariel M. A. Kennedy H. Wang X. J. (2015). A large-scale circuit mechanism for hierarchical dynamical processing in the primate cortex. Neuron 88, 419–431. doi: 10.1016/j.neuron.2015.09.008, PMID: 26439530 PMC4630024

[ref36] Chen N. Sugihara H. Sur M. (2015). An acetylcholine-activated microcircuit drives temporal dynamics of cortical activity. Nat. Neurosci. 18, 892–902. doi: 10.1038/nn.4002, PMID: 25915477 PMC4446146

[ref37] Cocchi L. Sale M. V. L L. G. Bell P. T. Nguyen V. T. Zalesky A.. (2016). A hierarchy of timescales explains distinct effects of local inhibition of primary visual cortex and frontal eye fields. elife 5:e15252. doi: 10.7554/eLife.15252, PMID: 27596931 PMC5012863

[ref38] Coen-Cagli R. Kohn A. Schwartz O. (2015). Flexible gating of contextual influences in natural vision. Nat. Neurosci. 18, 1648–1655. doi: 10.1038/nn.4128, PMID: 26436902 PMC4624479

[ref39] Cortay V. Delaunay D. Patti D. Gautier E. Doerflinger N. Giroud P.. (2020). Radial migration dynamics is modulated in a laminar and area-specific manner during primate Corticogenesis. Frontiers in Cell Developmental Biology 8:588814. doi: 10.3389/fcell.2020.588814, PMID: 33178700 PMC7596244

[ref40] Croner L. J. Albright T. D. (1997). Image segmentation enhances discrimination of motion in visual noise. Vis. Res. 37, 1415–1427. doi: 10.1016/s0042-6989(96)00299-4, PMID: 9205705

[ref41] Croner L. J. Albright T. D. (1999). Segmentation by color influences responses of motion-sensitive neurons in the cortical middle temporal visual area. J. Neurosci. 19, 3935–3951. doi: 10.1523/jneurosci.19-10-03935.1999, PMID: 10234024 PMC6782728

[ref42] de Lange F. P. Heilbron M. Kok P. (2018). How Do expectations shape perception? Trends Cogn. Sci. 22, 764–779. doi: 10.1016/j.tics.2018.06.002 30122170

[ref43] Dehay C. Kennedy H. Kosik K. S. (2015). The outer subventricular zone and primate-specific cortical complexification. Neuron 85, 683–694. doi: 10.1016/j.neuron.2014.12.060, PMID: 25695268

[ref44] D'Souza R. D. Wang Q. X. Ji W. Q. Meier A. M. Kennedy H. Knoblauch K.. (2022). Hierarchical and nonhierarchical features of the mouse visual cortical network. Nat. Commun. 13:503. doi: 10.1038/s41467-022-28035-y, PMID: 35082302 PMC8791996

[ref45] Eradath M. K. Pinsk M. A. Kastner S. (2021). A causal role for the pulvinar in coordinating task-independent cortico-cortical interactions. Journal of Comparative Neurology ePub 529, 3772–3784. doi: 10.1002/cne.25193, PMID: 34013540 PMC8556184

[ref46] Esmailpour H. Raman R. Vogels R. (2022). Inferior temporal cortex leads prefrontal cortex in response to a violation of a learned sequence. Cereb. Cortex 33, 3124–3141. doi: 10.1093/cercor/bhac265, PMID: 35780398

[ref47] Ferrer J. M. Price D. J. Blakemore C. (1988). The organization of corticocortical projections from area 17 to area 18 of the cat's visual cortex. Proc. R. Soc. Lond. Ser. B Biol. Sci. 233, 77–98. doi: 10.1098/rspb.1988.0013, PMID: 2895934

[ref48] Ferro D. van Kempen J. Boyd M. Panzeri S. Thiele A. (2021). Directed information exchange between cortical layers in macaque V1 and V4 and its modulation by selective attention. Proce. Natl. Acad. Sci. U. S. A. 118:e2022097118. doi: 10.1073/pnas.2022097118, PMID: 33723059 PMC8000025

[ref49] Feuerriegel D. Vogels R. Kovács G. (2021). Evaluating the evidence for expectation suppression in the visual system. Neurosci. Biobehav. Rev. 126, 368–381. doi: 10.1016/j.neubiorev.2021.04.002, PMID: 33836212

[ref50] Fiser A. Mahringer D. Oyibo H. K. Petersen A. V. Leinweber M. Keller G. B. (2016). Experience-dependent spatial expectations in mouse visual cortex. Nat. Neurosci. 19, 1658–1664. doi: 10.1038/nn.4385, PMID: 27618309

[ref51] Fitzgerald T. H. Valentin A. Selway R. Richardson M. P. (2013). Cross-frequency coupling within and between the human thalamus and neocortex. Front. Hum. Neurosci. 7:84. doi: 10.3389/fnhum.2013.00084, PMID: 23532592 PMC3607084

[ref52] Freiwald W. A. Tsao D. Y. (2010). Functional compartmentalization and viewpoint generalization within the macaque face-processing system. Science 330, 845–851. doi: 10.1126/science.1194908, PMID: 21051642 PMC3181095

[ref53] Fries P. (2005). A mechanism for cognitive dynamics: neuronal communication through neuronal coherence. Trends Cogn. Sci. 9, 474–480. doi: 10.1016/j.tics.2005.08.011 16150631

[ref54] Fries P. (2015). Rhythms for cognition: communication through coherence. Neuron 88, 220–235. doi: 10.1016/j.neuron.2015.09.034, PMID: 26447583 PMC4605134

[ref55] Friston K. (2005). A theory of cortical responses. Philosophical transactions of the Royal Society of London. Series B: Biological Sciences 360, 815–836. doi: 10.1098/rstb.2005.1622, PMID: 15937014 PMC1569488

[ref56] Friston K. (2008). Hierarchical models in the brain. PLoS Comput. Biol. 4:e1000211. doi: 10.1371/journal.pcbi.1000211, PMID: 18989391 PMC2570625

[ref57] Friston K. J. Bastos A. M. Oswal A. van Wijk B. Richter C. Litvak V. (2014). Granger causality revisited. NeuroImage 101, 796–808. doi: 10.1016/j.neuroimage.2014.06.062, PMID: 25003817 PMC4176655

[ref58] Friston K. Kiebel S. (2009). Predictive coding under the free-energy principle. Philosophical Transactions of the Royal Society of London. Series B: Biological Science 364, 1211–1221. doi: 10.1098/rstb.2008.0300, PMID: 19528002 PMC2666703

[ref59] Fujita I. Fujita T. (1996). Intrinsic connections in the macaque inferior temporal cortex. J. Comp. Neurol. 368, 467–486. doi: 10.1002/(SICI)1096-9861(19960513)368:4<467::AID-CNE1>3.0.CO;2-2, PMID: 8744437

[ref60] Gauthier B. Eger E. Hesselmann G. Giraud A. L. Kleinschmidt A. (2012). Temporal tuning properties along the human ventral visual stream. J. Neurosci. 32, 14433–14441. doi: 10.1523/jneurosci.2467-12.2012, PMID: 23055513 PMC6622391

[ref61] Gegenfurtner K. R. Kiper D. C. Beusmans J. M. H. Carandini M. Zaidi Q. Movshon J. A. (1994). Chromatic properties of neurons in macaque MT. Vis. Neurosci. 11, 455–466. doi: 10.1017/s095252380000239x 8038122

[ref62] Gibson J. J. (1979). The Ecological Approach to Visual Perception. Boston: Houghton Mifflin.

[ref63] Gieselmann M. A. Thiele A. (2022). Stimulus dependence of directed information exchange between cortical layers in macaque V1. elife 11:21. doi: 10.7554/eLife.62949.sa2 PMC8916775 35274614

[ref64] Gray C. M. McCormick D. A. (1996). Chattering cells: superficial pyramidal neurons contributing to the generation of synchronous oscillations in the visual cortex. Science 274, 109–113. doi: 10.1126/science.274.5284.109, PMID: 8810245

[ref65] Gregory R. L. (1980). Perceptions as hypotheses. Philos. Trans. R. Soc. Lond. B Biol. Sci. 290, 181–197. doi: 10.1098/rstb.1980.0090 6106237

[ref66] Grimaldi P. Saleem K. S. Tsao D. (2016). Anatomical connections of the functionally defined "face patches" in the macaque monkey. Neuron 90, 1325–1342. doi: 10.1016/j.neuron.2016.05.009, PMID: 27263973 PMC5573145

[ref67] Grothe I. Neitzel S. D. Mandon S. Kreiter A. K. (2012). Switching neuronal inputs by differential modulations of gamma-band phase-coherence. J. Neurosci. 32, 16172–16180. doi: 10.1523/jneurosci.0890-12.2012, PMID: 23152601 PMC6794021

[ref68] Gur M. Kagan I. Snodderly D. M. (2005). Orientation and direction selectivity of neurons in V1 of alert monkeys: functional relationships and laminar distributions. Cereb. Cortex 15, 1207–1221. doi: 10.1093/cercor/bhi003, PMID: 15616136

[ref69] Gur M. Snodderly D. M. (2008). Physiological differences between neurons in layer 2 and layer 3 of primary visual cortex (V1) of alert macaque monkeys. J. Physiol. 586, 2293–2306. doi: 10.1113/jphysiol.2008.151795, PMID: 18325976 PMC2479568

[ref70] Harris J. A. Mihalas S. Hirokawa K. E. Whitesell J. D. Choi H. Bernard A.. (2019). Hierarchical organization of cortical and thalamic connectivity. Nature 575, 195–202. doi: 10.1038/s41586-019-1716-z, PMID: 31666704 PMC8433044

[ref71] Hasson U. Yang E. Vallines I. Heeger D. J. Rubin N. (2008). A hierarchy of temporal receptive windows in human cortex. J. Neurosci. 28, 2539–2550. doi: 10.1523/jneurosci.5487-07.2008, PMID: 18322098 PMC2556707

[ref72] Hawken M. J. Parker A. J. Lund J. S. (1988). Laminar organization and contrast selectivity of direction selective cells in the striate cortex of the old-world monkey. J. Neurosci. 8, 3541–3548. doi: 10.1523/jneurosci.08-10-03541.1988, PMID: 3193169 PMC6569616

[ref73] Heilbron M. Chait M. (2018). Great expectations: is there evidence for predictive coding in auditory cortex? Neuroscience 389, 54–73. doi: 10.1016/j.neuroscience.2017.07.061 28782642

[ref74] Helmholtz H. (1860/1962). Handbuch der Physiologischen Optik, Vol 3. (English Trans: JPC Southall, Ed). New York: Dover.

[ref75] Hesse J. K. Tsao D. Y. (2020). The macaque face patch system: a turtle's underbelly for the brain. Nat. Rev. Neurosci. 21, 695–716. doi: 10.1038/s41583-020-00393-w, PMID: 33144718

[ref76] Hogendoorn H. Burkitt A. N. (2018). Predictive coding of visual object position ahead of moving objects revealed by time-resolved EEG decoding. NeuroImage 171, 55–61. doi: 10.1016/j.neuroimage.2017.12.063, PMID: 29277651

[ref77] Hohwy J. Roepstorff A. Friston K. (2008). Predictive coding explains binocular rivalry: an epistemological review. Cognition 108, 687–701. doi: 10.1016/j.cognition.2008.05.010, PMID: 18649876

[ref78] Honey C. J. Thesen T. Donner T. H. Silbert L. J. Carlson C. E. Devinsky O.. (2012). Slow cortical dynamics and the accumulation of information over long timescales. Neuron 76, 423–434. doi: 10.1016/j.neuron.2012.08.011, PMID: 23083743 PMC3517908

[ref79] Horwitz G. D. Albright T. D. (2005). Paucity of chromatic linear motion detectors in macaque V1. J. Vis. 5, 525–533. doi: 10.1167/5.6.4, PMID: 16097865

[ref80] Hu J. M. Roe A. W. (2022). Functionally specific and sparse domain-based micro-networks in monkey V1 and V2. Curr. Biol. 32:2797. doi: 10.1016/j.cub.2022.04.095 35623347

[ref81] Iacaruso M. F. Gasler I. T. Hofer S. B. (2017). Synaptic organization of visual space in primary visual cortex. Nature 547, 449–452. doi: 10.1038/nature23019, PMID: 28700575 PMC5533220

[ref82] Ichinohe N. Borra E. Rockland K. (2012). Distinct feedforward and intrinsic neurons in posterior inferotemporal cortex revealed by in vivo connection imaging. Sci. Rep. 2:934. doi: 10.1038/srep00934, PMID: 23226832 PMC3515805

[ref83] Issa E. B. Cadieu C. F. DiCarlo J. J. (2018). Neural dynamics at successive stages of the ventral visual stream are consistent with hierarchical error signals. elife 7:24. doi: 10.7554/eLife.42870, PMID: 30484773 PMC6296785

[ref84] Issa E. B. DiCarlo J. J. (2012). Precedence of the eye region in neural processing of faces. J. Neurosci. 32, 16666–16682. doi: 10.1523/jneurosci.2391-12.2012, PMID: 23175821 PMC3542390

[ref85] Jehee J. F. Ballard D. H. (2009). Predictive feedback can account for biphasic responses in the lateral geniculate nucleus. PLoS Comput. Biol. 5:e1000373. doi: 10.1371/journal.pcbi.1000373, PMID: 19412529 PMC2670540

[ref86] Jia X. Smith M. A. Kohn A. (2011). Stimulus selectivity and spatial coherence of gamma components of the local field potential. J. Neurosci. 31, 9390–9403. doi: 10.1523/jneurosci.0645-11.2011, PMID: 21697389 PMC3133446

[ref87] Jordan R. Keller G. B. (2020). Opposing influence of top-down and bottom-up input on excitatory layer 2/3 neurons in mouse primary visual cortex. Neuron 108, 1194–1206.e1195. doi: 10.1016/j.neuron.2020.09.024 33091338 PMC7772056

[ref88] Kaiser D. Quek G. L. Cichy R. M. Peelen M. V. (2019). Object vision in a structured world. Trends Cogn. Sci. 23, 672–685. doi: 10.1016/j.tics.2019.04.013, PMID: 31147151 PMC7612023

[ref89] Kaliukhovich D. A. Vogels R. (2011). Stimulus repetition probability does not affect repetition suppression in macaque inferior temporal cortex. Cereb. Cortex 21, 1547–1558. doi: 10.1093/cercor/bhq207, PMID: 21097993

[ref90] Kanai R. Komura Y. Shipp S. Friston K. (2015). Cerebral hierarchies: predictive processing, precision and the pulvinar. Philos. Trans. R. Soc. Lond. B Biol. Sci. 370:20140169. doi: 10.1098/rstb.2014.0169, PMID: 25823866 PMC4387510

[ref91] Kaposvari P. Kumar S. Vogels R. (2018). Statistical learning signals in macaque inferior temporal cortex. Cereb. Cortex 28, 250–266. doi: 10.1093/cercor/bhw374, PMID: 27909007

[ref92] Katsanevaki C. Bastos A. M. Cagnan H. Bosman C. A. Friston K. J. Fries P. (2023). Attentional effects on local V1 microcircuits explain selective V1-V4 communication. NeuroImage 281:120375. doi: 10.1016/j.neuroimage.2023.120375 37714390

[ref93] Keller G. B. Bonhoeffer T. Hubener M. (2012). Sensorimotor mismatch signals in primary visual cortex of the behaving mouse. Neuron 74, 809–815. doi: 10.1016/j.neuron.2012.03.040, PMID: 22681686

[ref94] Keller G. B. Mrsic-Flogel T. D. (2018). Predictive processing: a canonical cortical computation. Neuron 100, 424–435. doi: 10.1016/j.neuron.2018.10.003, PMID: 30359606 PMC6400266

[ref95] Kogo N. Trengove C. (2015). Is predictive coding theory articulated enough to be testable? Front. Comput. Neurosci. 9:111. doi: 10.3389/fncom.2015.00111, PMID: 26441621 PMC4561670

[ref96] Kok P. Brouwer G. J. van Gerven M. A. J. de Lange F. P. (2013). Prior expectations bias sensory representations in visual cortex. J. Neurosci. 33, 16275–16284. doi: 10.1523/jneurosci.0742-13.2013, PMID: 24107959 PMC6618350

[ref97] Kreiter A. K. (2006). How do we model attention-dependent signal routing? Neural Netw. 19, 1443–1444. doi: 10.1016/j.neunet.2006.09.005, PMID: 17064880

[ref98] Leinweber M. Ward D. R. Sobczak J. M. Attinger A. Keller G. B. (2017). A sensorimotor circuit in mouse cortex for visual flow predictions. Neuron 95, 1420–1432.e1425. doi: 10.1016/j.neuron.2017.08.036 28910624

[ref99] Leopold D. A. Logothetis N. K. (1996). Activity changes in early visual cortex reflect monkeys' percepts during binocular rivalry. Nature 379, 549–553. doi: 10.1038/379549a0, PMID: 8596635

[ref100] Levitt J. B. Yoshioka T. Lund J. S. (1994). Intrinsic cortical connections in macaque visual area V2: evidence for interaction between different functional streams. J. Comp. Neurol. 342, 551–570. doi: 10.1002/cne.903420405, PMID: 8040365

[ref101] Liu T. S. (2019). Feature-based attention: effects and control. Curr. Opin. Psychol. 29, 187–192. doi: 10.1016/j.copsyc.2019.03.013, PMID: 31015180 PMC6756988

[ref102] Livingstone M. S. Hubel D. H. (1984a). Anatomy and physiology of a color system in the primate visual cortex. J. Neurosci. 4, 309–356. doi: 10.1523/jneurosci.04-01-00309.1984, PMID: 6198495 PMC6564760

[ref103] Livingstone M. S. Hubel D. H. (1984b). Specificity of intrinsic connections in primate primary visual cortex. J. Neurosci. 4, 2830–2835. doi: 10.1523/jneurosci.04-11-02830.1984, PMID: 6209365 PMC6564722

[ref104] Lochmann T. Blanche T. J. Butts D. A. (2013). Construction of direction selectivity through local energy computations in primary visual cortex. PLoS One 8:e58666. doi: 10.1371/journal.pone.0058666, PMID: 23554913 PMC3598900

[ref105] Logothetis N. K. Schall J. D. (1989). Neuronal correlates of subjective visual perception. Science 245, 761–763. doi: 10.1126/science.2772635 2772635

[ref106] Lopes da Silva F. H. Vos J. E. Mooibroek J. Van Rotterdam A. (1980). Relative contributions of intracortical and thalamo-cortical processes in the generation of alpha rhythms, revealed by partial coherence analysis. Electroencephalogr. Clin. Neurophysiol. 50, 449–456. doi: 10.1016/0013-4694(80)90011-5, PMID: 6160987

[ref107] Luck S. J. Chelazzi L. Hillyard S. A. Desimone R. (1997). Neural mechanisms of spatial selective attention in areas V1, V2 and V4 of macaque visual cortex. J. Neurophysiol. 77, 24–42. doi: 10.1152/jn.1997.77.1.24, PMID: 9120566

[ref108] Lund J. S. Hawken M. J. Parker A. J. (1988). Local circuit neurons of macaque monkey striate cortex: II. Neurons of laminae 5B and 6. J. Comp. Neurol. 276, 1–29. doi: 10.1002/cne.902760102, PMID: 2461395

[ref109] Lund J. S. Hendrickson A. E. Ogren M. P. Tobin E. A. (1981). Anatomical organization of primate visual cortex area VII. J. Comp. Neurol. 202, 19–45. doi: 10.1002/cne.902020104, PMID: 6793644

[ref110] Lund J. S. Wu C. Q. (1997). Local circuit neurons of macaque monkey striate cortex: IV. Neurons of laminae 1-3A. J. Comp. Neurol. 384, 109–126. doi: 10.1002/(sici)1096-9861(19970721)384:1<109::aid-cne7>3.0.co;2-5, PMID: 9214543

[ref111] Lund J. S. Yoshioka T. Levitt J. B. (1993). Comparison of intrinsic connectivity in different areas of macaque monkey cerebral cortex. Cereb. Cortex 3, 148–162. doi: 10.1093/cercor/3.2.148, PMID: 8490320

[ref112] Markov N. T. Vezoli J. Chameau P. Falchier A. Quilodran R. Huissoud C.. (2014). Anatomy of hierarchy: feedforward and feedback pathways in macaque visual cortex. J. Comp. Neurol. 522, 225–259. doi: 10.1002/cne.23458, PMID: 23983048 PMC4255240

[ref113] Maunsell J. H. Treue S. (2006). Feature-based attention in visual cortex. Trends Neurosci. 29, 317–322. doi: 10.1016/j.tins.2006.04.001 16697058

[ref114] Meyer T. Olson C. R. (2011). Statistical learning of visual transitions in monkey inferotemporal cortex. Proceedings of the National Academy of Sciences USA 108, 19401–19406. doi: 10.1073/pnas.1112895108 PMC3228439 22084090

[ref115] Meyer T. Ramachandran S. Olson C. R. (2014). Statistical learning of serial visual transitions by neurons in monkey inferotemporal cortex. J. Neurosci. 34, 9332–9337. doi: 10.1523/jneurosci.1215-14.2014, PMID: 25009266 PMC4087210

[ref116] Michalareas G. Vezoli J. van Pelt S. Schoffelen J. M. Kennedy H. Fries P. (2016). Alpha-Beta and Gamma rhythms subserve feedback and feedforward influences among human visual cortical areas. Neuron 89, 384–397. doi: 10.1016/j.neuron.2015.12.018, PMID: 26777277 PMC4871751

[ref117] Moran J. Desimone R. (1985). Selective attention gates visual processing in the extrastriate cortex. Science 229, 782–784. doi: 10.1126/science.4023713 4023713

[ref118] Movshon J. A. Newsome W. T. (1996). Visual response properties of striate cortical neurons projecting to area MT in macaque monkeys. J. Neurosci. 16, 7733–7741. doi: 10.1523/jneurosci.16-23-07733.1996 8922429 PMC6579106

[ref119] Mumford D. (1992). On the computational architecture of the neocortex. II. The role of cortico-cortical loops. Biol. Cybern. 66, 241–251. doi: 10.1007/BF00198477 1540675

[ref120] Murray R. F. Adams W. J. (2019). Visual perception and natural illumination. Curr. Opin. Behav. Sci. 30, 48–54. doi: 10.1016/j.cobeha.2019.06.001

[ref121] Murray J. D. Bernacchia A. Freedman D. J. Romo R. Wallis J. D. Cai X.. (2014). A hierarchy of intrinsic timescales across primate cortex. Nat. Neurosci. 17, 1661–1663. doi: 10.1038/nn.3862, PMID: 25383900 PMC4241138

[ref122] Muzzu T. Saleem A. B. (2021). Feature selectivity can explain mismatch signals in mouse visual cortex. Cell Rep. 37:109772. doi: 10.1016/j.celrep.2021.109772, PMID: 34610298 PMC8655498

[ref123] Nakamura H. Gattass R. Desimone R. Ungerleider L. G. (1993). The modular organization of projections from areas V1 and V2 to areas V4 and TEO in macaques. J. Neurosci. 13, 3681–3691. doi: 10.1523/jneurosci.13-09-03681.1993, PMID: 7690064 PMC6576450

[ref124] Nassi J. J. Callaway E. M. (2007). Specialized circuits from primary visual cortex to V2 and area MT. Neuron 55, 799–808. doi: 10.1016/j.neuron.2007.07.037, PMID: 17785186 PMC2727861

[ref125] Niell C. M. Stryker M. P. (2010). Modulation of visual responses by behavioral state in mouse visual cortex. Neuron 65, 472–479. doi: 10.1016/j.neuron.2010.01.033, PMID: 20188652 PMC3184003

[ref126] Ninomiya T. Sawamura H. Inoue K. Takada M. (2011). Differential architecture of multisynaptic geniculo-cortical pathways to V4 and MT. Cereb. Cortex 21, 2797–2808. doi: 10.1093/cercor/bhr078, PMID: 21515714

[ref127] O’Toole S. M. Oyibo H. K. Keller G. B. (2023). Molecularly targetable cell types in mouse visual cortex have distinguishable prediction error responses. Neuron. 111, 2918–2928.e2918. doi: 10.1016/j.neuron.2023.08.015 37708892

[ref128] Onorato I. Neuenschwander S. Hoy J. Lima B. Rocha K. S. Broggini A. C.. (2020). A distinct class of bursting neurons with strong gamma synchronization and stimulus selectivity in monkey V1. Neuron 105, 180–197.e185. doi: 10.1016/j.neuron.2019.09.039 31732258

[ref129] Peter A. Uran C. Klon-Lipok J. Roese R. van Stijn S. Barnes W.. (2019). Surface color and predictability determine contextual modulation of V1 firing and gamma oscillations. elife 8:e42101. doi: 10.7554/eLife.42101 30714900 PMC6391066

[ref130] Phillips W. A. (2017). Cognitive functions of intracellular mechanisms for contextual amplification. Brain Cogn. 112, 39–53. doi: 10.1016/j.bandc.2015.09.005, PMID: 26428863

[ref131] Piasini E. Soltuzu L. Muratore P. Caramellino R. Vinken K. Op de Beeck H.. (2021). Temporal stability of stimulus representation increases along rodent visual cortical hierarchies. Nat. Commun. 12:4448. doi: 10.1038/s41467-021-24456-3, PMID: 34290247 PMC8295255

[ref132] Pinto L. Goard M. J. Estandian D. Xu M. Kwan A. C. Lee S. H.. (2013). Fast modulation of visual perception by basal forebrain cholinergic neurons. Nat. Neurosci. 16, 1857–1863. doi: 10.1038/nn.3552, PMID: 24162654 PMC4201942

[ref133] Quax S. Jensen O. Tiesinga P. (2017). Top-down control of cortical gamma-band communication via pulvinar induced phase shifts in the alpha rhythm. PLoS Comput. Biol. 13:e1005519. doi: 10.1371/journal.pcbi.1005519, PMID: 28472057 PMC5436894

[ref134] Ramachandran S. Meyer T. Olson C. R. (2016). Prediction suppression in monkey inferotemporal cortex depends on the conditional probability between images. J. Neurophysiol. 115, 355–362. doi: 10.1152/jn.00091.2015, PMID: 26581864 PMC4760508

[ref135] Ramachandran S. Meyer T. Olson C. R. (2017). Prediction suppression and surprise enhancement in monkey inferotemporal cortex. J. Neurophysiol. 118, 374–382. doi: 10.1152/jn.00136.2017, PMID: 28424293 PMC5501919

[ref136] Rao R. P. Ballard D. H. (1999). Predictive coding in the visual cortex: a functional interpretation of some extra-classical receptive-field effects. Nat. Neurosci. 2, 79–87. doi: 10.1038/4580, PMID: 10195184

[ref137] Ray S. Maunsell J. H. (2010). Differences in gamma frequencies across visual cortex restrict their possible use in computation. Neuron 67, 885–896. doi: 10.1016/j.neuron.2010.08.004, PMID: 20826318 PMC3001273

[ref138] Reynolds J. H. Chelazzi L. Desimone R. (1999). Competitive mechanisms subserve attention in macaque areas V2 and V4. J. Neurosci. 19, 1736–1753. doi: 10.1523/JNEUROSCI.19-05-01736.1999, PMID: 10024360 PMC6782185

[ref139] Richter C. G. Thompson W. H. Bosman C. A. Fries P. (2017). Top-down Beta enhances bottom-up gamma. J. Neurosci. 37, 6698–6711. doi: 10.1523/jneurosci.3771-16.2017 28592697 PMC5508256

[ref140] Roberts M. J. Lowet E. Brunet N. M. Ter Wal M. Tiesinga P. Fries P.. (2013). Robust gamma coherence between macaque V1 and V2 by dynamic frequency matching. Neuron 78, 523–536. doi: 10.1016/j.neuron.2013.03.003, PMID: 23664617

[ref141] Robinson D. L. Bowman E. M. Kertzman C. (1995). Covert orienting of attention in macaques. II. Contributions of parietal cortex. J. Neurophysiol. 74, 698–712. doi: 10.1152/jn.1995.74.2.698 7472375

[ref142] Rockland K. S. (1992a). Configuration, in serial reconstruction, of individual axons projecting from area V2 to V4 in the macaque monkey. Cereb. Cortex 2, 353–374. doi: 10.1093/cercor/2.5.353, PMID: 1384848

[ref143] Rockland K. S. (1992b). Laminar distribution of neurons projecting from area V1 to V2 in macaque and squirrel monkeys. Cereb. Cortex 2, 38–47. doi: 10.1093/cercor/2.1.38, PMID: 1378768

[ref144] Rockland K. S. Pandya D. N. (1979). Laminar origins and terminations of cortical connections of the occipital lobe in the rhesus monkey. Brain Res. 179, 3–20. doi: 10.1016/0006-8993(79)90485-2, PMID: 116716

[ref145] Saalmann Y. B. Pinsk M. A. Wang L. Li X. Kastner S. (2012). The pulvinar regulates information transmission between cortical areas based on attention demands. Science 337, 753–756. doi: 10.1126/science.1223082, PMID: 22879517 PMC3714098

[ref146] Sajedin A. Menhaj M. B. Vahabie A. H. Panzeri S. Esteky H. (2019). Cholinergic modulation promotes attentional modulation in primary visual cortex – a modeling study. Sci. Rep. 9:20186. doi: 10.1038/s41598-019-56608-3, PMID: 31882838 PMC6934489

[ref147] Saleem A. B. Ayaz A. Jeffery K. J. Harris K. D. Carandini M. (2013). Integration of visual motion and locomotion in mouse visual cortex. Nat. Neurosci. 16, 1864–1869. doi: 10.1038/nn.3567, PMID: 24185423 PMC3926520

[ref148] Sawatari A. Callaway E. M. (2000). Diversity and cell type specificity of local excitatory connections to neurons in layer 3B of monkey primary visual cortex. Neuron 25, 459–471. doi: 10.1016/s0896-6273(00)80908-3, PMID: 10719899

[ref149] Schwiedrzik C. M. Freiwald W. A. (2017). High-level prediction signals in a low-level area of the macaque face-processing hierarchy. Neuron 96, 89–97.e4. doi: 10.1016/j.neuron.2017.09.007, PMID: 28957679 PMC5757317

[ref150] Shipp S. (2003). The functional logic of cortico-pulvinar connections. Philos. Trans. R. Soc. Lond. B Biol. Sci. 358, 1605–1624. doi: 10.1098/rstb.2002.1213, PMID: 14561322 PMC1693262

[ref151] Shipp S. (2004). The brain circuitry of attention. Trends Cogn. Sci. 8, 223–230. doi: 10.1016/j.tics.2004.03.004 15120681

[ref152] Shipp S. (2016). Neural elements for predictive coding. Front. Psychol. 7:1792. doi: 10.3389/fpsyg.2016.01792, PMID: 27917138 PMC5114244

[ref153] Shipp S. Adams R. A. Friston K. J. (2013). Reflections on agranular architecture: predictive coding in the motor cortex. Trends Neurosci. 36, 706–716. doi: 10.1016/j.tins.2013.09.004, PMID: 24157198 PMC3858810

[ref154] Shipp S. Friston K. (2023). “Predictive coding: forward and backward connectivity” in The Cerebral Cortex and Thalamus. eds. Usrey W. M. Sherman S. M. (New York: Oxford University Press)

[ref155] Shipp S. Grant S. (1991). Organization of reciprocal connections between area 17 and the lateral suprasylvian area of cat visual cortex. Vis. Neurosci. 6, 339–355. doi: 10.1017/s095252380000657x, PMID: 1711892

[ref156] Shipp S. Moutoussis K. Adams D. Zeki S. (2009). Feature binding in the feedback layers of area V2. Cereb. Cortex 19, 2230–2239. doi: 10.1093/cercor/bhn243, PMID: 19153106 PMC2742589

[ref157] Shipp S. Zeki S. (1989). The organization of connections between areas V5 and V1 in macaque monkey visual cortex. Eur. J. Neurosci. 1, 309–332. doi: 10.1111/j.1460-9568.1989.tb00798.x, PMID: 12106142

[ref158] Shipp S. Zeki S. (2002). The functional organisation of area V2. I: specialisation of function across stripes and layers. Vis. Neurosci. 19, 187–210. doi: 10.1017/s0952523802191164 12385630

[ref159] Siegle J. H. Jia X. Durand S. Gale S. Bennett C. Graddis N.. (2021). Survey of spiking in the mouse visual system reveals functional hierarchy. Nature 592, 86–92. doi: 10.1038/s41586-020-03171-x, PMID: 33473216 PMC10399640

[ref160] Sincich L. C. Blasdel G. G. (2001). Oriented axon projections in primary visual cortex of the monkey. J. Neurosci. 21, 4416–4426. doi: 10.1523/jneurosci.21-12-04416.2001, PMID: 11404428 PMC6762731

[ref161] Siu C. Balsor J. Merlin S. Federer F. Angelucci A. (2021). A direct interareal feedback-to-feedforward circuit in primate visual cortex. Nat. Commun. 12:4911. doi: 10.1038/s41467-021-24928-6, PMID: 34389710 PMC8363744

[ref162] Sohn W. Papathomas T. V. Blaser E. Vidnyanszky Z. (2004). Object-based cross-feature attentional modulation from color to motion. Vis. Res. 44, 1437–1443. doi: 10.1016/j.visres.2003.12.010, PMID: 15066402

[ref163] Solomon S. S. Tang H. Sussman E. Kohn A. (2021). Limited evidence for sensory prediction error responses in visual cortex of macaques and humans. Cereb. Cortex 31, 3136–3152. doi: 10.1093/cercor/bhab014, PMID: 33683317 PMC8599921

[ref164] Spratling M. W. (2016). A review of predictive coding algorithms. Brain Cogn. 112, 92–97. doi: 10.1016/j.bandc.2015.11.003 26809759

[ref165] Steele G. E. Weller R. E. Cusick C. G. (1991). Cortical connections of the caudal subdivision of the dorsolateral area (V4) in monkeys. J. Comp. Neurol. 306, 495–520. doi: 10.1002/cne.903060312 1713928

[ref166] Steinmetz M. A. Constantinidis C. (1995). Neurophysiological evidence for a role of posterior parietal cortex in redirecting visual attention. Cereb. Cortex 5, 448–456. doi: 10.1093/cercor/5.5.448 8547791

[ref167] Summerfield C. Trittschuh E. H. Monti J. M. Mesulam M. M. Egner T. (2008). Neural repetition suppression reflects fulfilled perceptual expectations. Nat. Neurosci. 11, 1004–1006. doi: 10.1038/nn.2163, PMID: 19160497 PMC2747248

[ref168] Sutton R. S. Barto A. G. (1998). Reinforcement Learning. Cambridge, MA: The MIT Press.

[ref169] Tang H. Buia C. Madhavan R. Crone N. E. Madsen J. R. Anderson W. S.. (2014). Spatiotemporal dynamics underlying object completion in human ventral visual cortex. Neuron 83, 736–748. doi: 10.1016/j.neuron.2014.06.017, PMID: 25043420 PMC4134509

[ref170] Tasic B. Yao Z. Graybuck L. T. Smith K. A. Nguyen T. N. Bertagnolli D.. (2018). Shared and distinct transcriptomic cell types across neocortical areas. Nature 563, 72–78. doi: 10.1038/s41586-018-0654-5, PMID: 30382198 PMC6456269

[ref171] Taubert J. Wardle S. G. Tardiff C. T. Koele E. A. Kumar S. Messinger A.. (2022). The cortical and subcortical correlates of face pareidolia in the macaque brain. Soc. Cogn. Affect. Neurosci. 17, 965–976. doi: 10.1093/scan/nsac031, PMID: 35445247 PMC9629476

[ref172] Thiele A. Dobkins K. R. Albright T. D. (1999). The contribution of color to motion processing in macaque middle temporal area. J. Neurosci. 19, 6571–6587. doi: 10.1523/jneurosci.19-15-06571.1999, PMID: 10414985 PMC6782820

[ref173] Thomas R. M. De Sanctis T. Gazzola V. Keysers C. (2018). Where and how our brain represents the temporal structure of observed action. NeuroImage 183, 677–697. doi: 10.1016/j.neuroimage.2018.08.056, PMID: 30165253 PMC6215330

[ref174] Thompson P. Burr D. (2009). Visual aftereffects. Curr. Biol. 19, R11–R14. doi: 10.1016/j.cub.2008.10.014 19138580

[ref175] Todorova G. K. Pollick F. E. Muckli L. (2021). Special treatment of prediction errors in autism spectrum disorder. Neuropsychologia 163:108070. doi: 10.1016/j.neuropsychologia.2021.108070, PMID: 34695420

[ref176] Ungerleider L. G. Galkin T. W. Desimone R. Gattass R. (2008). Cortical connections of area V4 in the macaque. Cereb. Cortex 18, 477–499. doi: 10.1093/cercor/bhm061 17548798

[ref177] Uran C. Peter A. Lazar A. Barnes W. Klon-Lipok J. Shapcott K. A.. (2022). Predictive coding of natural images by V1 firing rates and rhythmic synchronization. Neuron 110, 1240–1257.e8. doi: 10.1016/j.neuron.2022.01.002, PMID: 35120628 PMC8992798

[ref178] Valverde F. (1978). The organization of area 18 in the monkey. A Golgi study. Anat. Embryol. 154, 305–334. doi: 10.1007/BF00345659 101094

[ref179] van Kerkoerle T. Self M. W. Dagnino B. Gariel-Mathis M. A. Poort J. van der Togt C.. (2014). Alpha and gamma oscillations characterize feedback and feedforward processing in monkey visual cortex. Proceedings of the National Academy of Sciences USA 111, 14332–14341. doi: 10.1073/pnas.1402773111, PMID: 25205811 PMC4210002

[ref180] van Pelt S. Heil L. Kwisthout J. Ondobaka S. van Rooij I. Bekkering H. (2016). Beta- and gamma-band activity reflect predictive coding in the processing of causal events. Soc. Cogn. Affect. Neurosci. 11, 973–980. doi: 10.1093/scan/nsw017, PMID: 26873806 PMC4884316

[ref181] Vasilevskaya A. Widmer F. C. Keller G. B. Jordan R. (2023). Locomotion-induced gain of visual responses cannot explain visuomotor mismatch responses in layer 2/3 of primary visual cortex. Cell Rep. 42:112096. doi: 10.1016/j.celrep.2023.112096, PMID: 36821437 PMC9945359

[ref9001] Vezoli J. Magrou L. Goebel R. Wang X. J. Knoblauch K. Vinck M.. (2021). Cortical hierarchy, dual counterstream architecture and the importance of top-down generative networks. Neuroimage. 225:117479. doi: 10.1016/j.neuroimage.2020.117479 33099005 PMC8244994

[ref182] Vinken K. Op de Beeck H. P. Vogels R. (2018). Face repetition probability does not affect repetition suppression in macaque Inferotemporal cortex. J. Neurosci. 38, 7492–7504. doi: 10.1523/jneurosci.0462-18.2018, PMID: 30030399 PMC6596142

[ref183] Vinken K. Vogels R. (2017). Adaptation can explain evidence for encoding of probabilistic information in macaque inferior temporal cortex. Curr. Biol. 27, R1210–R1212. doi: 10.1016/j.cub.2017.09.018, PMID: 29161556

[ref184] Vogels R. (2016). Sources of adaptation of inferior temporal cortical responses. Cortex 80, 185–195. doi: 10.1016/j.cortex.2015.08.024 26518166

[ref185] Walsh K. S. McGovern D. P. Clark A. O'Connell R. G. (2020). Evaluating the neurophysiological evidence for predictive processing as a model of perception. Ann. N. Y. Acad. Sci. 1464, 242–268. doi: 10.1111/nyas.14321, PMID: 32147856 PMC7187369

[ref186] Wang X. J. (2010). Neurophysiological and computational principles of cortical rhythms in cognition. Physiol. Rev. 90, 1195–1268. doi: 10.1152/physrev.00035.2008, PMID: 20664082 PMC2923921

[ref187] Wang W. Jones H. E. Andolina I. M. Salt T. E. Sillito A. M. (2006). Functional alignment of feedback effects from visual cortex to thalamus. Nat. Neurosci. 9, 1330–1336. doi: 10.1038/nn1768, PMID: 16980966

[ref188] Weiler S. Nilo D. G. Bonhoeffer T. Hubener M. Rose T. Scheuss V. (2023). Functional and structural features of L2/3 pyramidal cells continuously covary with pial depth in mouse visual cortex. Cerebral Cortex ePub 33, 3715–3733. doi: 10.1093/cercor/bhac303, PMID: 36017976 PMC10068292

[ref189] Weilnhammer V. Fritsch M. Chikermane M. Eckert A. L. Kanthak K. Stuke H.. (2021). An active role of inferior frontal cortex in conscious experience. Curr. Biol. 31, 2868–2880.e2868. doi: 10.1016/j.cub.2021.04.043 33989530

[ref190] Wiser A. K. Callaway E. M. (1996). Contributions of individual layer 6 pyramidal neurons to local circuitry in macaque primary visual cortex. J. Neurosci. 16, 2724–2739. doi: 10.1523/JNEUROSCI.16-08-02724.1996, PMID: 8786448 PMC6578755

[ref191] Wiser A. K. Callaway E. M. (1997). Ocular dominance columns and local projections of layer 6 pyramidal neurons in macaque primary visual cortex. Vis. Neurosci. 14, 241–251. doi: 10.1017/S095252380001138X, PMID: 9147477

[ref192] Yabuta N. H. Callaway E. M. (1998). Functional streams and local connections of layer 4C neurons in primary visual cortex of the macaque monkey. J. Neurosci. 18, 9489–9499. doi: 10.1523/jneurosci.18-22-09489.1998, PMID: 9801386 PMC6792868

[ref193] Yoshioka T. Blasdel G. G. Levitt J. B. Lund J. S. (1996). Relation between patterns of intrinsic lateral connectivity, ocular dominance, and cytochrome oxidase-reactive regions in macaque monkey striate cortex. Cereb. Cortex 6, 297–310. doi: 10.1093/cercor/6.2.297, PMID: 8670658

[ref194] Yoshioka T. Levitt J. B. Lund J. S. (1992). Intrinsic lattice connections of macaque monkey visual cortical area V4. J. Neurosci. 12, 2785–2802. doi: 10.1523/jneurosci.12-07-02785.1992, PMID: 1377236 PMC6575826

[ref195] Yu J. Ferster D. (2013). Functional coupling from simple to complex cells in the visually driven cortical circuit. J. Neurosci. 33, 18855–18866. doi: 10.1523/jneurosci.2665-13.2013, PMID: 24285892 PMC3841452

[ref196] Yukie M. Iwai E. (1985). Laminar origin of direct projection from cortex area V1 to V4 in the rhesus monkey. Brain Res. 346, 383–386. doi: 10.1016/0006-8993(85)90875-3, PMID: 4052788

[ref197] Zeki S. Shipp S. (1989). Modular connections between areas V2 and V4 of macaque monkey visual cortex. Eur. J. Neurosci. 1, 494–506. doi: 10.1111/j.1460-9568.1989.tb00356.x, PMID: 12106135

[ref198] Zhang X. L. Qiu J. Zhang Y. Y. Han S. H. Fang F. (2014). Misbinding of color and motion in human visual cortex. Curr. Biol. 24, 1354–1360. doi: 10.1016/j.cub.2014.04.045, PMID: 24856212

[ref199] Zhang S. Xu M. Kamigaki T. Hoang Do J. P. Chang W. C. Jenvay S.. (2014). Selective attention. Long-range and local circuits for top-down modulation of visual cortex processing. Science 345, 660–665. doi: 10.1126/science.1254126, PMID: 25104383 PMC5776147

[ref200] Zhou H. Schafer R. J. Desimone R. (2016). Pulvinar-cortex interactions in vision and attention. Neuron 89, 209–220. doi: 10.1016/j.neuron.2015.11.034, PMID: 26748092 PMC4723640

[ref201] Zmarz P. Keller G. B. (2016). Mismatch receptive fields in mouse visual cortex. Neuron 92, 766–772. doi: 10.1016/j.neuron.2016.09.057, PMID: 27974161

